# Synthesis and structure of a new thiazoline-based palladium(II) complex that promotes cytotoxicity and apoptosis of human promyelocytic leukemia HL-60 cells

**DOI:** 10.1038/s41598-020-73488-0

**Published:** 2020-10-07

**Authors:** Javier Espino, Elena Fernández-Delgado, Samuel Estirado, Felipe de la Cruz-Martinez, Sergio Villa-Carballar, Emilio Viñuelas-Zahínos, Francisco Luna-Giles, José A. Pariente

**Affiliations:** 1 Department of Physiology (Neuroimmunophysiology and Chrononutrition Research Group), Faculty of Science, University of Extremadura, 06006 Badajoz, Spain; 2 Departament of Organic and Inorganic Chemistry (Chemistry of Coordination Research Group), Faculty of Science, University of Extremadura, Badajoz, Spain

**Keywords:** Cancer, Drug discovery

## Abstract

Cisplatin is one of the most widely used chemotherapeutic agents in the treatment of different tumors but has high toxicity and side effects. Therefore, the synthesis of new chemotherapeutic agents is necessary, so that they are effective in the treatment of cancer while avoiding such toxicity. In this study, we have synthesized and characterized a palladium(II) complex, [PdCl_2_(µ-PyTT)_2_]Cl_2_·4H_2_O (PdPyTT), with 2-(2-pyridyl)imine-N-(2-thiazolin-2-yl)thiazolidine (PyTT) as a ligand; besides, its cytotoxicity and pro-apoptotic capacity was tested in human promyelocytic leukemia HL-60 cell line. Similar to cisplatin, PdPyTT produced a time- and dose-dependent decrease in cell viability. Additionally, the palladium complex increased both the proportion of cells with apoptotic morphology and the activation of caspase-3 and -9. PdPyTT, like cisplatin, also increased intracellular ROS production and DNA oxidative damage. Therefore, our findings demonstrated the promising application of palladium(II) complexes as novel anti-leukemic agents.

## Introduction

Cisplatin and its derivatives are common chemotherapeutic agents used to treat different cancers since they can activate cell death mechanisms in cancer cells^1–4^. However, cisplatin produces serious side effects as it also affects healthy cells^5–8^ and, in addition, certain cancers may also acquire resistance to this drug. Therefore, developing novel platinum(II)-based compounds is warranted to broaden the palette of anticancer drugs. Given the similarity of coordination chemistry, organometallic palladium(II) complexes have been also synthesized in recent years. In fact, different mononuclear and dinuclear palladium(II) complexes with promising antitumor activity have been previously described^9–11^.

New coordination complexes are obtained by adding bioactive ligands, e.g., heterocycles bearing donor atoms, into their structure. Thiazolines are thiazole-derived heterocycles bearing a single double bond as well as nitrogen and sulphur atoms. In this sense, it has been shown that 2-thiazoline-based oligomers induce cytotoxicity in HPAC, PC-3, and HCT-116 human cell lines (pancreatic, prostate and colon cancer, respectively)^12^. Likewise, it has been reported that new compounds designed around a thiazolidine ring^13^ or bearing a thiazolidine-2,4-dione^14^ promote cell cycle arrest and/or mitochondrial apoptosis in different cancer cell lines, including human promyelocytic leukemia HL-60 cells.

Furthermore, previous studies have reported several organometallic complexes with pro-apoptotic properties. Thus, it has been reported that copper(II) complexes bearing a mixed 2-amino-2-thiazoline ligand inside the coordination sphere exhibit antiproliferative (cytostatic and cytotoxic) activity against a panel of tumor cell lines (including HeLa, L929, HT-29 and T47D)^15^, as well as antitumor and anti-inflammatory activities in in vivo preparations of leukemic rats^16^. Besides, platinum(II) and palladium(II) complexes containing 2-mercaptothiazoline have been shown to produce remarkable cytotoxic actions against hepatoma cells^17^. In this line, we have also recently demonstrated that platinum(II) and palladium(II) complexes containing a thiazoline derivative ligand reduced proliferation capacity and induced apoptosis in both colon HT-29 and lymphoma U-937 cell lines^18^.

Herein, we have synthesized and characterized a palladium(II) complex coordinated with PyTT (2-(2-pyridyl)imine-N-(2-thiazolin-2-yl)thiazolidine), a thiazoline derivative ligand. In addition, we also aimed to study the potential cytotoxic and pro-apoptotic capacity of the complex PdPyTT in human promyelocytic leukemia HL-60 cells.

## Results and discussion

### Chemical synthesis

**Scheme 1:**
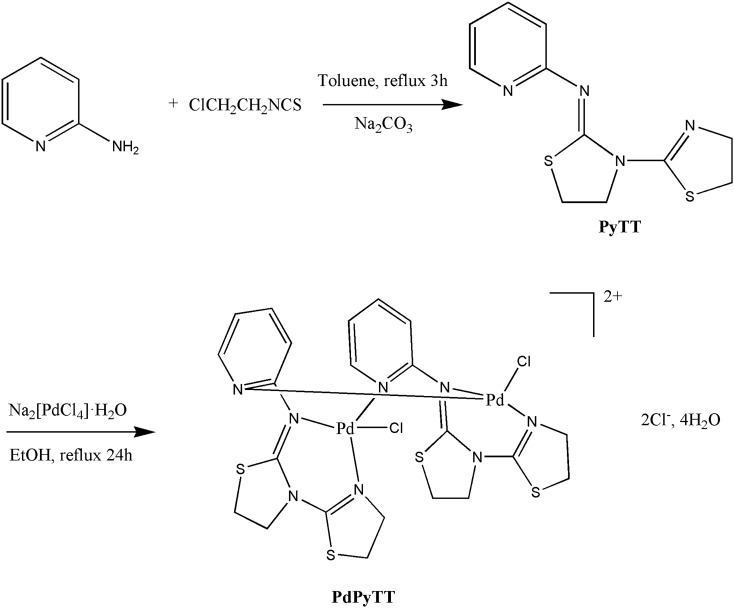
Preparation of PyTT and PdPyTT.

The reaction of ligand PyTT with Na_2_[PdCl_4_]·H_2_O in EtOH produced the complex PdPyTT from the evaporation of a filtered EtOH solution. The molecular structure of PdPyTT was obtained by means of single crystal X-ray diffraction. A general scheme which represents the strategy employed for the synthesis of the ligand and metal complex is illustrated in Scheme 1.

### Crystal structure of PdPyTT

**Figure 1:**
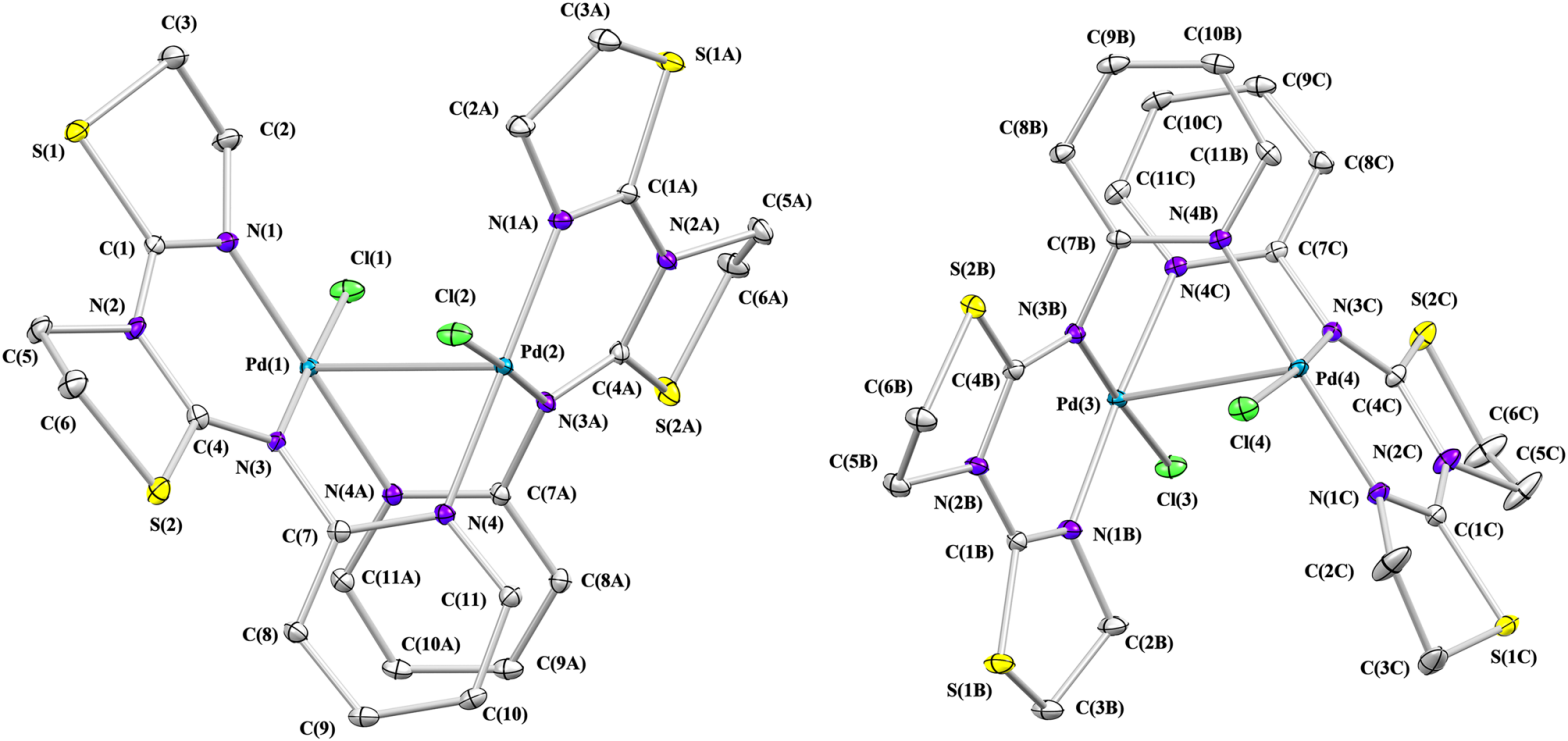
Molecular structure of the independent cationic complexes [PdCl_2_(µ-PyTT)_2_]^2+^ in PdPyTT. Hydrogen atoms were omitted for clarity.

The single crystal X-ray diffraction analysis has revealed that PdPyTT crystallizes in the monoclinic system with two independent binuclear palladium cationic complexes, four chlorine ions and eight crystallization water molecules in the asymmetric units and, therefore, the complex salt can be formulated as [PdCl_2_(µ-PyTT)_2_]Cl_2_·4H_2_O. The molecular structure of the two types of independent cationic complexes is shown in Fig. 1 and selected bond lengths and angles are given in Table 1. As can be seen, the coordination environments for metallic centres are the same, showing a slightly distorted square-pyramidal geometry (τ = 0.03–0.07 for palladium(II) centres)^19^. The organic ligand PyTT is tridentate bridging two palladium ions, linking to one palladium metal by means of imino nitrogen atom and thiazoline nitrogen atom and the other palladium by means of pyridine nitrogen atom, thus forming five and six membered chelate rings. A terminal chloro ligand and the second palladium atom completing five-coordination around each metal. The Pd–Pd, Pd–Cl and Pd–N bond lengths are comparable to those observed for the unique binuclear palladium complex with a chromophore group Pd_2_Cl_2_N_6_, dichloro-bis(1,3-diphenyl-5-(benzothiazol-2-yl)formazanato)-di-palladium(II)^20^ obtained from Cambridge Structural Database (CSD, Version v5.41, Nov 2019)^21^. Moreover, bonding parameters of organic ligand are similar to those reported for other metal complexes with PyTT^22–26^.

**Table 1:** Selected bond lengths (Å) and angles (°) for PdPyTT.

Pd(1)-Pd(2)	3.114(1)	Pd(1)-Cl(1)	2.308(1)
Pd(1)-N(1)	1.996(2)	Pd(1)-N(3)	2.050(2)
Pd(1)-N(4A)	2.020(2)	Pd(2)-Cl(2)	2.300(1)
Pd(2)-N(1A)	1.992(2)	Pd(2)-N(3A)	2.044(2)
Pd(2)-N(4)	2.022(2)	Pd(3)-Pd(4)	3.112(1)
Pd(3)-Cl(3)	2.309(1)	Pd(3)-N(1B)	2.006(2)
Pd(3)-N(3B)	2.040(2)	Pd(3)-N(4C)	2.040(2)
Pd(4)-Cl(4)	2.322(1)	Pd(4)-N(1C)	1.994(2)
Pd(4)-N(3C)	2.041(2)	Pd(4)-N(4B)	2.030(2)
Cl(1)-Pd(1)-Pd(2)	112.3(1)	Cl(1)-Pd(1)-N(1)	93.8(1)
Cl(1)-Pd(1)-N(3)	177.3(1)	Cl(1)-Pd(1)-N(4A)	86.8(1)
N(1)-Pd(1)-Pd(2)	101.6(1)	N(1)-Pd(1)-N(3)	88.8(1)
N(1)-Pd(1)-N(4A)	179.2(1)	N(3)-Pd(1)-Pd(2)	67.7(1)
N(3)-Pd(1)-N(4A)	90.6(1)	N(4A)-Pd(1)-Pd(2)	77.6(1)
Cl(2)-Pd(2)-Pd(1)	112.5(1)	Cl(2)-Pd(2)-N(1A)	93.5(1)
Cl(2)-Pd(2)-N(3A)	177.5(1)	Cl(2)-Pd(2)-N(4)	86.5(1)
N(1A)-Pd(2)-Pd(1)	102.1(1)	N(1A)-Pd(2)-N(3A)	88.8(1)
N(1A)-Pd(2)-N(4)	179.8(1)	N(3A)-Pd(2)-Pd(1)	68.0(1)
N(3A)-Pd(2)-N(4)	91.3(1)	N(4)-Pd(2)-Pd(1)	78.2(1)
Cl(3)-Pd(3)-Pd(4)	110.3(1)	Cl(3)-Pd(3)-N(1B)	94.5(1)
Cl(3)-Pd(3)-N(3B)	177.4(1)	Cl(3)-Pd(3)-N(4C)	86.6(1)
N(1B)-Pd(3)-Pd(4)	97.5(1)	N(1B)-Pd(3)-N(3B)	87.9(1)
N(1B)-Pd(3)-N(4C)	175.3(1)	N(3B)-Pd(3)-Pd(4)	68.2(1)
N(3B)-Pd(3)-N(4C)	90.9(1)	N(4C)-Pd(3)-Pd(4)	77.9(1)
Cl(4)-Pd(4)-Pd(3)	107.0(1)	Cl(4)-Pd(4)-N(1C)	94.3(1)
Cl(4)-Pd(4)-N(3C)	174.3(1)	Cl(4)-Pd(4)-N(4B)	86.2(1)
N(1C)-Pd(4)-Pd(3)	103.8(1)	N(1C)-Pd(4)-N(3C)	88.7(1)
N(1C)-Pd(4)-N(4B)	178.7(1)	N(3C)-Pd(4)-Pd(3)	67.6(1)
N(3C)-Pd(4)-N(4B)	90.9(1)	N(4B)-Pd(4)-Pd(3)	77.2(1)

Cationic dinuclear complexes are self-assembled by means of a non-conventional hydrogen bonding network of the type C–H···Cl–Pd and C–H···S (see Supplementary Table S1 online) to generate a 3D supramolecular metal organic matrix having helical channels along a-axis (see Supplementary Figure S1 online). The helical channels are defined with an approximate size of 8.33 × 4.92 Å acting as template for the helical water-chloride chain. These pores are filled by the four non-coordinated chlorine ion and the eight crystallization water molecules of the asymmetric unit resulting either left-handed as right-handed water-chloride helical chains in a 1:1 relation.

Water molecules and chlorine ions are self-assembled in the chain by means of a network of sixteen hydrogen bonding of which twelve are the type O–H···Cl^−^ and four of the type O–H···O in a helical arrangement. Inside the chain, four water molecules of eight of crystallization (O1W, O7W, O8W, O2W) act as donors of hydrogen participating in the formation of two hydrogen bonds, each of which donates two hydrogen (O1W, O2W) to two chloride anions and (O7W, O8W) to two water molecules, while the other four (O3W, O4W, O5W, O6W) are involved in three hydrogen bonds each donating two hydrogen to two chloride ions (O3W, O4W to Cl6, Cl7 and O5W, O6W to Cl5, Cl8) and accepting one hydrogen. The water-chloride chain can be described as an infinite sequence of four-membered ring (2Cl^−^ + 2H_2_O) and six-membered ring (2Cl^−^ + 4H_2_O) arranged alternately joined together sharing a chloride ion and a water molecule with a T4(2)6(2) topology derived of water clusters assembly^27^ so that the minimal repeating motif along the chain is {[(H_2_O)_8_Cl_4_]^4−^}_n_ (see Supplementary Figure S2 online).

The crystal packing between the water-chloride chain and metal–organic matrix is maintained by the formation of weak hydrogen bonds among some C–H atoms of pyridine, thiazoline and thiazolidine heterocycles of organic ligands and eight crystallization water of the chain, thereby generating fourteen C–H···O hydrogen bonds [average d(H···O) = 2.48 Å, average d(C···O) = 3.21 Å, average $\angle$(C–H···O) = 131°]t^28,29^ and four non coordinated chlorine ions producing eight C–H···Cl^−^ hydrogen bonds [average d(H··· Cl^−^) = 2,81 Å, average d(C··· Cl^−^) = 3,21 Å, average $\angle$(C–H··· Cl^−^) = 139.7°]^30,31^ (Table S1). Thus, these weak intermolecular interactions within the crystal of the type C–H···O and C–H···Cl^-^ between the water–chloride chain and bimetallic cationic complexes build up a stable supramolecular assembly in which the water-chloride chains (guest) are supported by a metal–organic framework acting as host.

### Spectroscopic studies

By comparison of the ^1^H-NMR spectral data for PyTT and PdPyTT (see Supplementary Figures S3–S4 online), all ^1^H-NMR signals in complex are slightly shifted to downfield with respect to the unbonded ligand.

The IR spectrum of PdPyTT (see Supplementary Figures S5–S6 online) showed a strong absorption at 1601 cm^−1^ corresponding to ν(C=N)_imine_ and W_1_[ν(C=N)] vibrations. These bands are shifted positively relative to the uncoordinated imine functions of the respective ligand (1590 cm^−1^). This feature is indicative for coordination through the imine and thiazoline nitrogen atoms^32^. Other relevant band is the one assignable to ν(H_2_O) stretching vibration at 3417 cm^−1^ due to crystallization water molecules in PdPyTT^26^. In the low-frequency region, the *C*_1_ symmetry of PdPyTT predicts the appearance of four bands assignable to Pd–ligand stretching vibrations. The ν(Pd-Cl) vibration is registered at 325 cm^−1^, which is in good agreement with literature data^33,34^. Finally, the ν(Pd–N) vibrations can be attributed to the bands at 465, 285 and 237 cm^−1^ for ν(M-N_imine_), ν(M-N_pyridine_) and ν(M-N_thiazoline_) vibrations, respectively^33–38^.

### Biological studies

**Figure 2:**
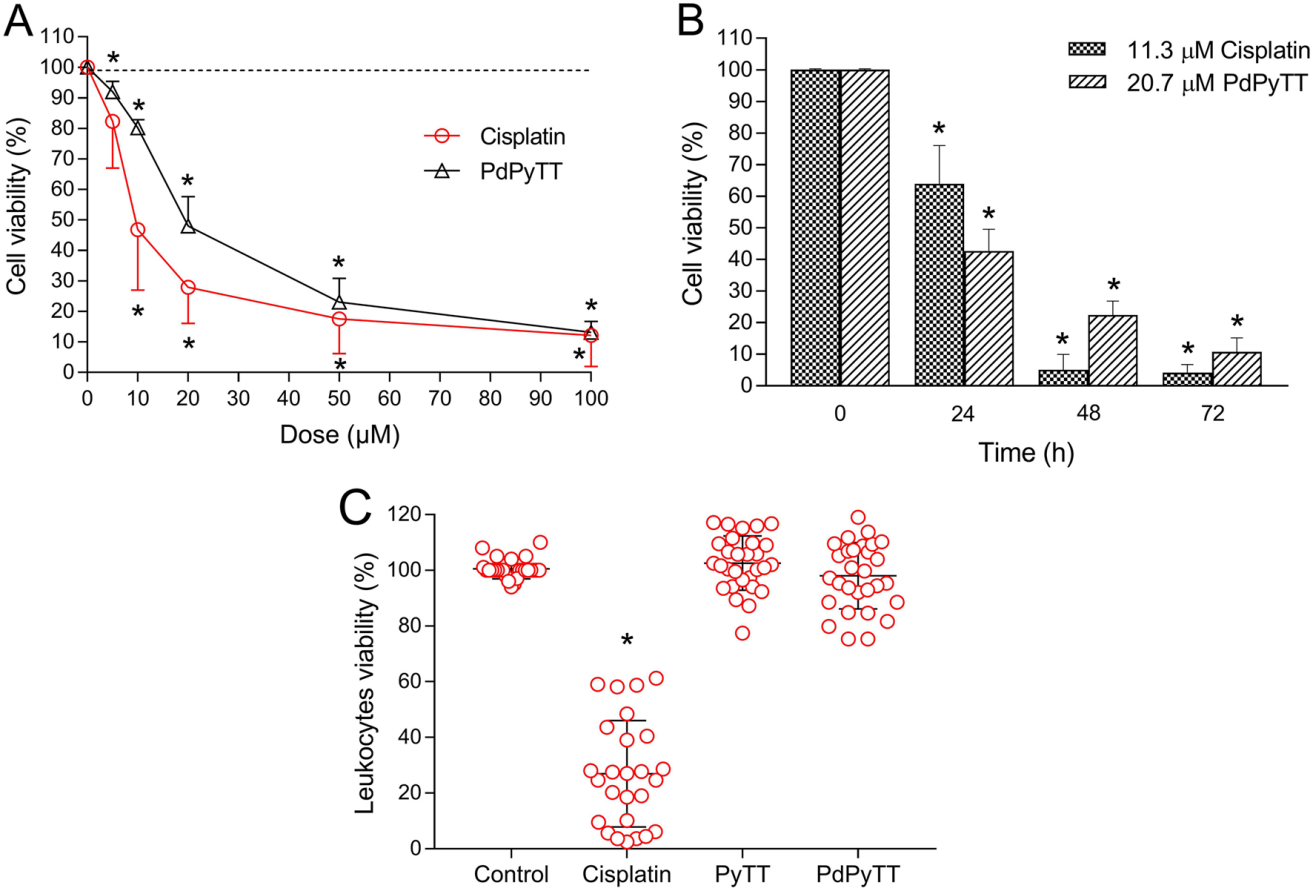
Dose- and time-response curves of chemotherapeutics on cell viability. (**A**) HL-60 cells were treated for 24 h with increasing concentrations (5, 10, 20, 50 and 100 µM) of cisplatin, the Pd(II) complex (PdPyTT), or the vehicle (DMSO, control). (**B**) HL-60 cells were treated with the IC_50_ of cisplatin (11.3 µM), of PdPyTT (20.7 µM), or the vehicle (DMSO, control) for 24, 48 and 72 h. (**C**) Freshly isolated human leukocytes were treated with 11.3 µM cisplatin, 20.7 µM PyTT, 20.7 µM PdPyTT, or the vehicle (DMSO, control) for 16 h. Values represent means ± S.D. of 6 independent experiments and are expressed as percentage of control values. *P < 0.05 compared to their corresponding control values.

We have studied the potential cytotoxic effect and the proapoptotic capacity of the complex PdPyTT in human promyelocytic leukemia HL-60 cells, comparing it with cisplatin, a well-known chemotherapeutic agent. First, we analysed the effect of the complex PdPyTT on cellular viability of HL-60 cell line and compared it with that induced by cisplatin. Figure 2 shows the viability of HL-60 cells in the presence of cisplatin and PdPyTT. Stimulation of cells with increasing concentrations (5, 10, 20, 50 and 100 µM) of cisplatin or PdPyTT for 24 h induced a remarkable dose-dependent decrease (Fig. 2A) in cell viability, reaching the minimum value of viability (12.1 ± 9.2% and 13.1 ± 3.6%, respectively, Fig. 2A) with the dose of 100 µM. The half maximal inhibitory concentrations (IC_50_) were 20.7 µM and 11.3 µM for PdPyTT and cisplatin, respectively. Figure 2B shows that the effects of both the complex PdPyTT and cisplatin were also time-dependent, although the levels of cell viability were almost completely suppressed after 48 h of incubation with cisplatin (5.1 ± 3.8%, Fig. 2B). In addition, the free ligand (PyTT) produced negligible effect on HL-60 cells viability (see Supplementary Figure S7 online), except for the highest dose (100 µM) and after 72 h of incubation at 20.7 µM (dose comparable to IC_50_ of PdPyTT). Similar results were obtained in human histiocytic lymphoma U-937 cells. Both PdPyTT and cisplatin produced a dose-dependent decline in cell viability (see Supplementary Figure S8 online). The IC_50_ values in U-937 cells were 22.5 µM and 7.9 µM for PdPyTT and cisplatin, respectively. It is also important to point out that the complex PdPyTT (as well as the free ligand PyTT) was proved innocuous for freshly isolated human leukocytes, whilst cisplatin showed a high toxicity towards these healthy cells in which viability dropped by roughly 70% (Fig. 2C). Even though IC_50_ of PdPyTT (20.7 µM) was two-fold higher compared to that of cisplatin (11.3 µM), the palladium(II) complex proved to be harmless for healthy human leukocytes. These findings are in good agreement with the cytotoxic activities found for palladium(II) complexes containing bis(2-pyridylmethyl)amine or saccharinate (IC_50_ values ranging between 17.4 and 32.3 mM) in A549 lung carcinoma cells^39,40^, though extremely low IC_50_ values (< 1 µM) have been reported for other palladium(II) complexes in brain glioma, breast cancer and fibrosarcoma cell lines^41–43^.

**Figure 3:**
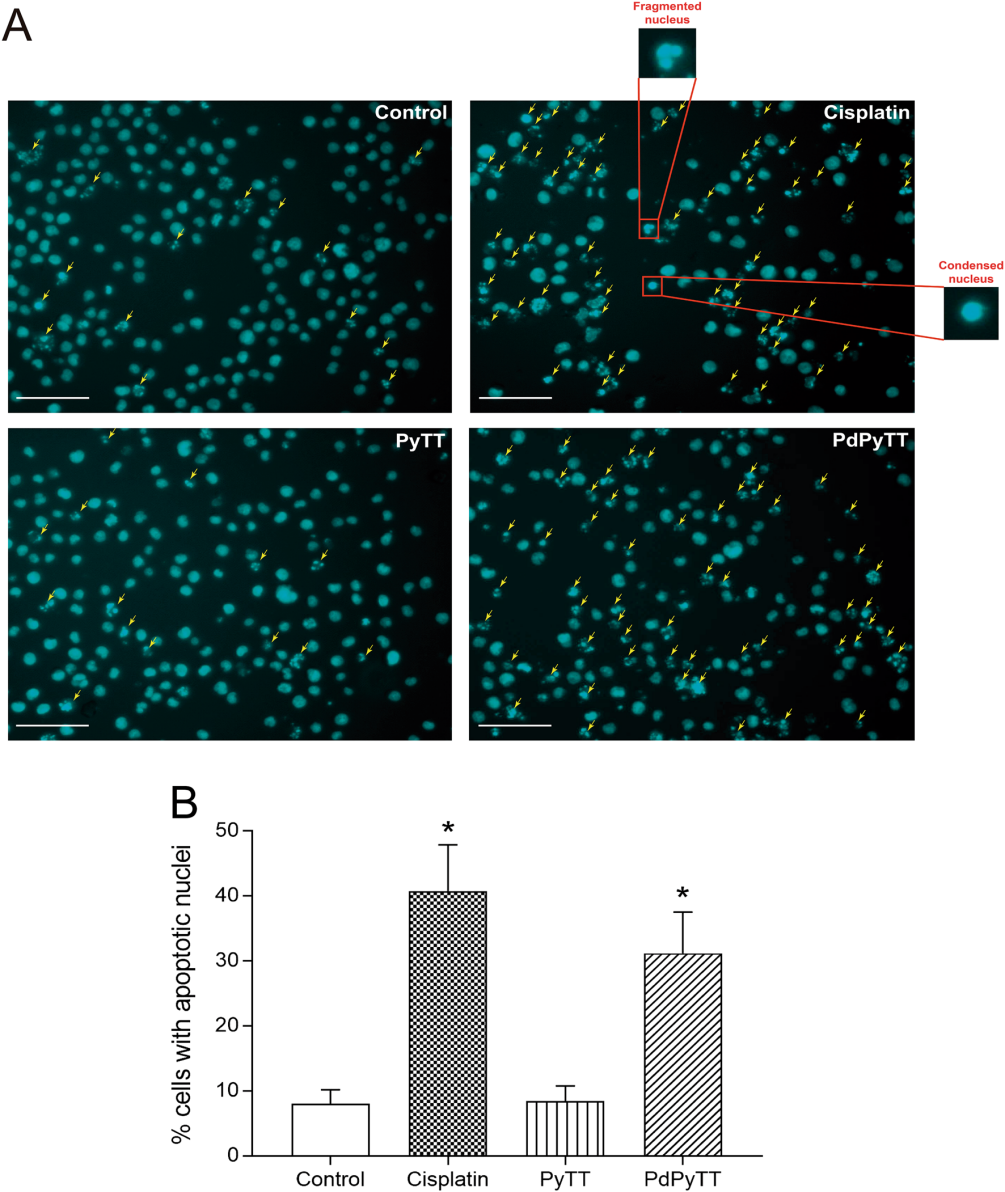
Effects of chemotherapeutics on apoptosis in HL-60 cells. Cells were treated with 11.3 µM cisplatin, 20.7 µM PyTT, 20.7 µM PdPyTT, or the vehicle (DMSO, control) for 24 h. (**A**) Hoechst 33342-stained cells were visualized with epifluorescence microscopy and representative images of each experimental condition are shown. The fraction of apoptotic nuclei was evaluated as indicated in the Materials and Methods section. Yellow arrows indicate apoptotic nuclei (i.e., fragmented or condensed). Both fragmented and condensed nuclei are also shown in greater detail in the magnifications. (**B**) Histograms show percentages of cells with apoptotic nucleus. Values represent means ± S.D. of 6 independent experiments. *P < 0.05 compared to control values. Scale bar: 100 µm.

**Figure 4:**
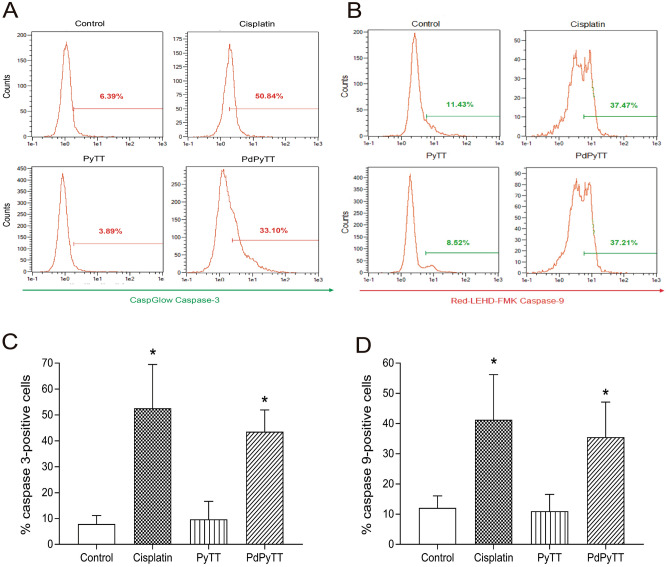
Effects of chemotherapeutics on caspases activation in HL-60 cells. Cells were treated with 11.3 µM cisplatin, 20.7 µM PyTT, 20.7 µM PdPyTT, or the vehicle (DMSO, control) for 24 h. Caspase-3 (**A**) and -9 (**B**) activation was measured by flow cytometry by means of CaspGLOW fluorescein active caspase-3 staining kit and Caspase-9 staining kit (Red-LEHD-FMK), respectively. Analysis was restricted to live cells that were detected by using the vital dye Hoechst 33258. Representative cytograms of each experimental condition are shown for both caspases. (**C** and **D**) Histograms show percentages of each cell population. Values represent means ± S.D. of 6 independent experiments. *P < 0.05 compared to control values.

To test whether the reduction in cell viability was associated with apoptosis induction, we analysed the percentage of apoptotic nuclei in Hoechst 33342-stained cells. As shown in Fig. 3, nuclear staining with Hoechst 33342 revealed that the treatment of HL-60 cells with 20.7 µM PdPyTT or 11.3 µM cisplatin for 24 h produced nuclear fragmentation and/or condensation (31.2 ± 6.3 and 40.7 ± 7.1% apoptotic cells for PdPyTT and cisplatin, respectively; Fig. 3B), thus indicating that the main form of cell death was apoptosis. These findings were corroborated by analysis of phosphatidylserine externalization in the presence of PI. In fact, the complex PdPyTT induced a significant rise in the proportion of annexin V-positive cells (see Supplementary Figure S9 online). Additionally, we also studied the activation of caspase-3 and caspase-9 in HL-60 cells in the presence of both chemotherapeutic agents. Treatment of cells with 20.7 µM PdPyTT or 11.3 µM cisplatin for 24 h gave rise to a clear increase in both caspase-3 (43.5 ± 15.7 and 52.6 ± 15.9% of caspase-3-positive cells for PdPyTT and cisplatin, respectively; Fig. 4A,C) and caspase-9 (35.5 ± 10.6 and 41.3 ± 13.9% of caspase-9-positive cells for PdPyTT and cisplatin, respectively; Fig. 4B,D). Furthermore, the free ligand (PyTT) again produced no effect on the percentage of apoptotic cells or the activation of caspases (Figs. 3 and 4, respectively).

**Figure 5:**
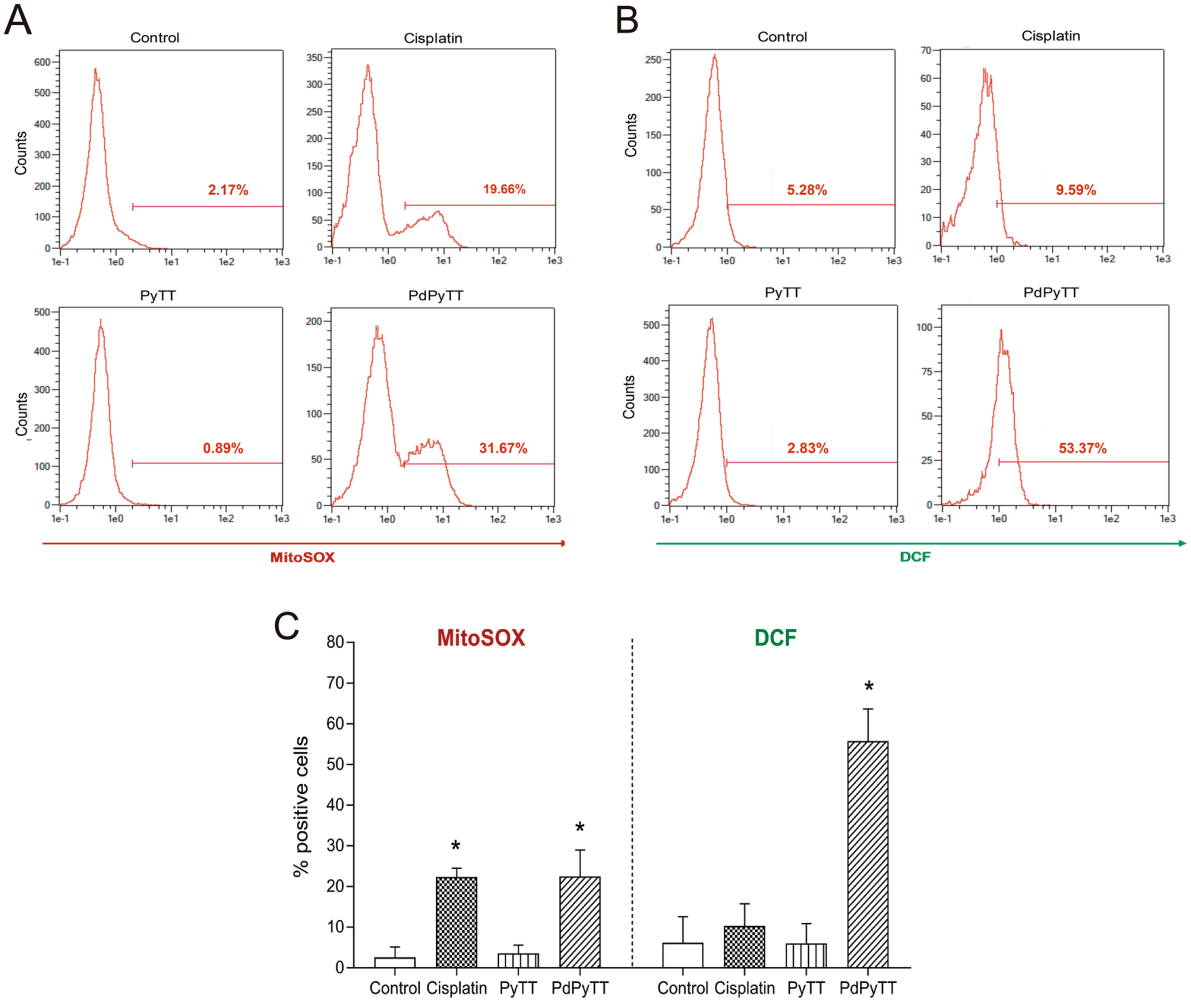
Effects of chemotherapeutics on the intracellular production of superoxide anion and reactive oxygen species (ROS) in HL-60 cells. Cells were treated with 11.3 µM cisplatin, 20.7 µM PyTT, 20.7 µM PdPyTT, or the vehicle (DMSO, control) for 24 h. The production of mitochondrial superoxide anion (**A**) and intracellular ROS (**B**) were determined by flow cytometry by means of MitoSOX and DCFH, respectively. Analysis was restricted to live cells that were detected by using the vital dye Hoechst 33,258. Representative cytograms of each experimental condition are shown. (**C**) Histograms show percentages of each cell population. Values represent means ± S.D. of 6 independent experiments. *P < 0.05 compared to control values.

These results were in line with increases in intracellular reactive oxygen species (ROS) production observed after treating HL-60 cells with the chemotherapeutic agents. As shown in Fig. 5, treatment of HL-60 cells with 20.7 µM PdPyTT or 11.3 µM cisplatin for 24 h caused a detectable rise in both mitochondrial superoxide anion and intracellular ROS generation as revealed by the increase in MitoSox (Fig. 5A,C) and dichlorofluorescein (DCF) fluorescence (Fig. 5B,C), respectively. The effect of cisplatin and PdPyTT on the localised production of mitochondrial superoxide anion was similar (22.3 ± 2.2 and 22.5 ± 6.4% of positive cells, respectively; Fig. 5C), while PdPyTT was more effective in inducing intracellular overproduction of ROS (55.7 ± 16.7% of positive cells; Fig. 5C) compared with the effect of cisplatin (10.3 ± 4.4% of positive cells; Fig. 5C). These findings suggest that the complex PdPyTT displayed potent antitumor actions that relied on its ability to trigger caspase-9-dependent mitochondrial apoptosis, on one hand, and to cause excessive formation of cytosolic and mitochondrial ROS, on the other hand. Like results on cell death, the free ligand (PyTT) did not produce any substantial effect on the production of intracellular ROS or mitochondrial superoxide anion (Fig. 5).

**Figure 6:**
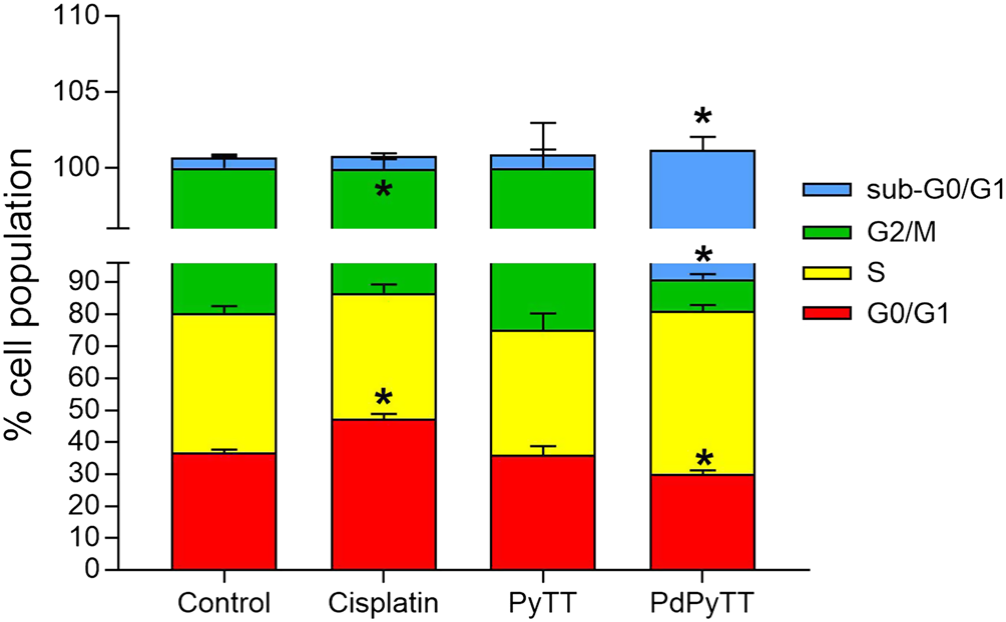
Chemotherapeutics disturbed cell cycle profile of HL-60 cells. Cells were treated with 11.3 µM cisplatin, 20.7 µM PyTT, 20.7 µM PdPyTT, or the vehicle (DMSO, control) for 24 h. Cell cycle distribution was determined by flow cytometry analysis using ethanol-fixed, propidium iodide-stained cells. Data from the cell cycle distribution were summarized and presented as percentage of cells. Cells with hypodiploid DNA content (sub-G1/G0 population) denote an increase in DNA fragmentation. Histograms show percentages of apoptotic cells. Values represent means ± S.D. of 6 independent experiments. *P < 0.05 compared to its corresponding control values.

Given the pro-apoptotic actions of the complex PdPyTT, its effect on cell cycle progression in HL-60 cells was anticipated and, therefore, a cell cycle distribution analysis was carried out. As shown in Fig. 6, HL-60 cells challenged with 20.7 µM PdPyTT exhibited a moderate, non-significant S phase arrest that was accompanied by noticeable (*P* < 0.05) diminution in the proportion of cells in both G0/G1 and G2/M subpopulations, while cells treated with 11.3 µM cisplatin depicted a significant (*P* < 0.05) accumulation of cells at G0/G1 phase and a remarkable (*P* < 0.05) reduction in cells for G2/M subpopulation. Besides, PdPyTT, but not cisplatin, caused substantial DNA fragmentation in HL-60 cells, as denoted by a significant increase in the subpopulation of cells with hypodiploid DNA content (sub-G1/G0; *P* < 0.05; Fig. 6). Again, the free ligand (PyTT) did not produce any significant change on cell cycle distribution (Fig. 6).

**Figure 7:**
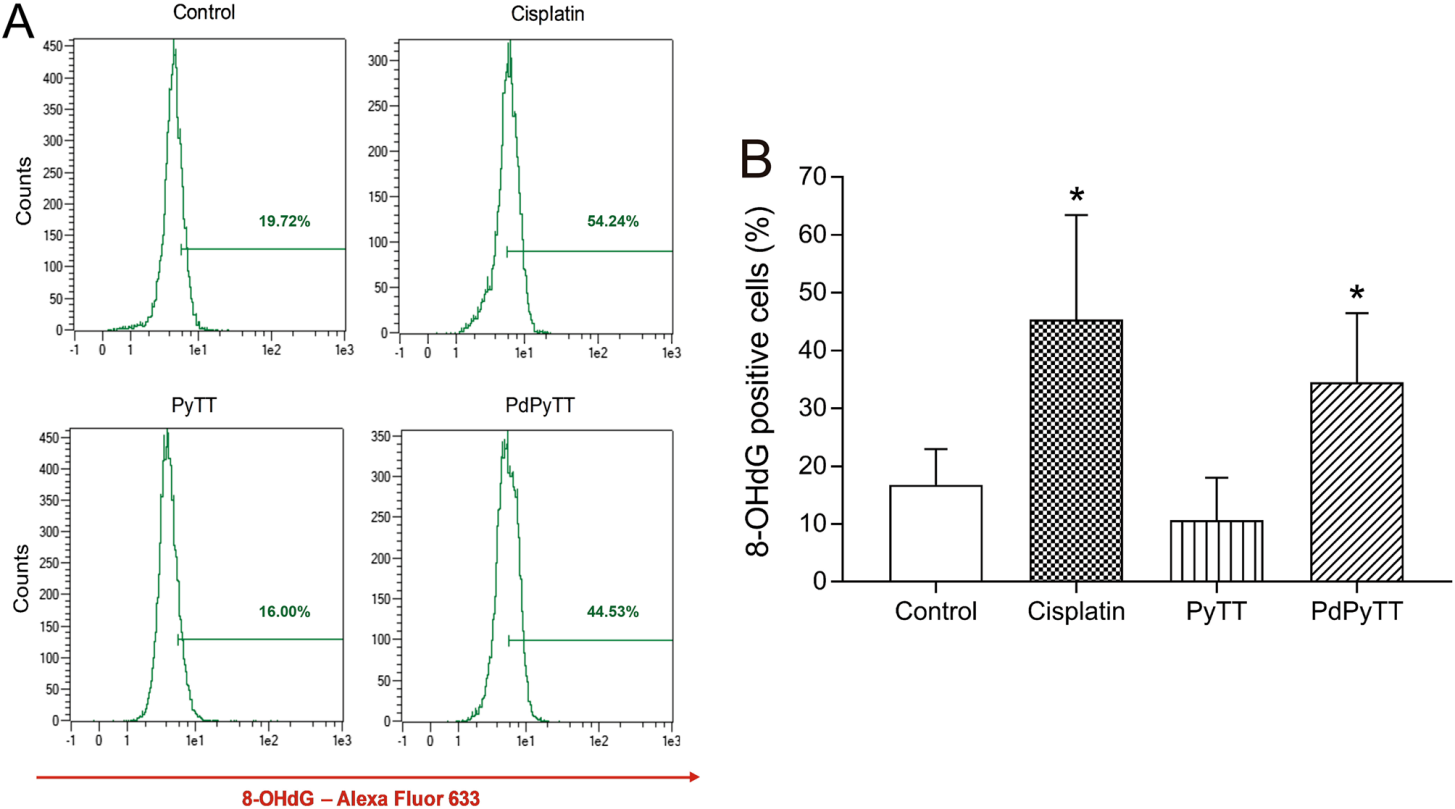
Effects of chemotherapeutics on DNA oxidative damage in HL-60 cells. Cells were treated with 11.3 µM cisplatin, 20.7 µM PyTT, 20.7 µM PdPyTT, or the vehicle (DMSO, control) for 24 h. (**A**) DNA oxidative damage was determined by flow cytometry in fixed cells labelled with anti-8-OHdG antibody and F(ab')2-goat anti-mouse IgG (H + L) secondary antibody, Alexa Fluor 633. Analysis was restricted to nucleated cells that were distinguished by staining cells with PI. Representative cytograms of each experimental condition are shown. (**B**) Histograms show percentages of each cell population. Values represent means ± S.D. of 6 independent experiments. *P < 0.05 compared to control values.

**Figure 8:**
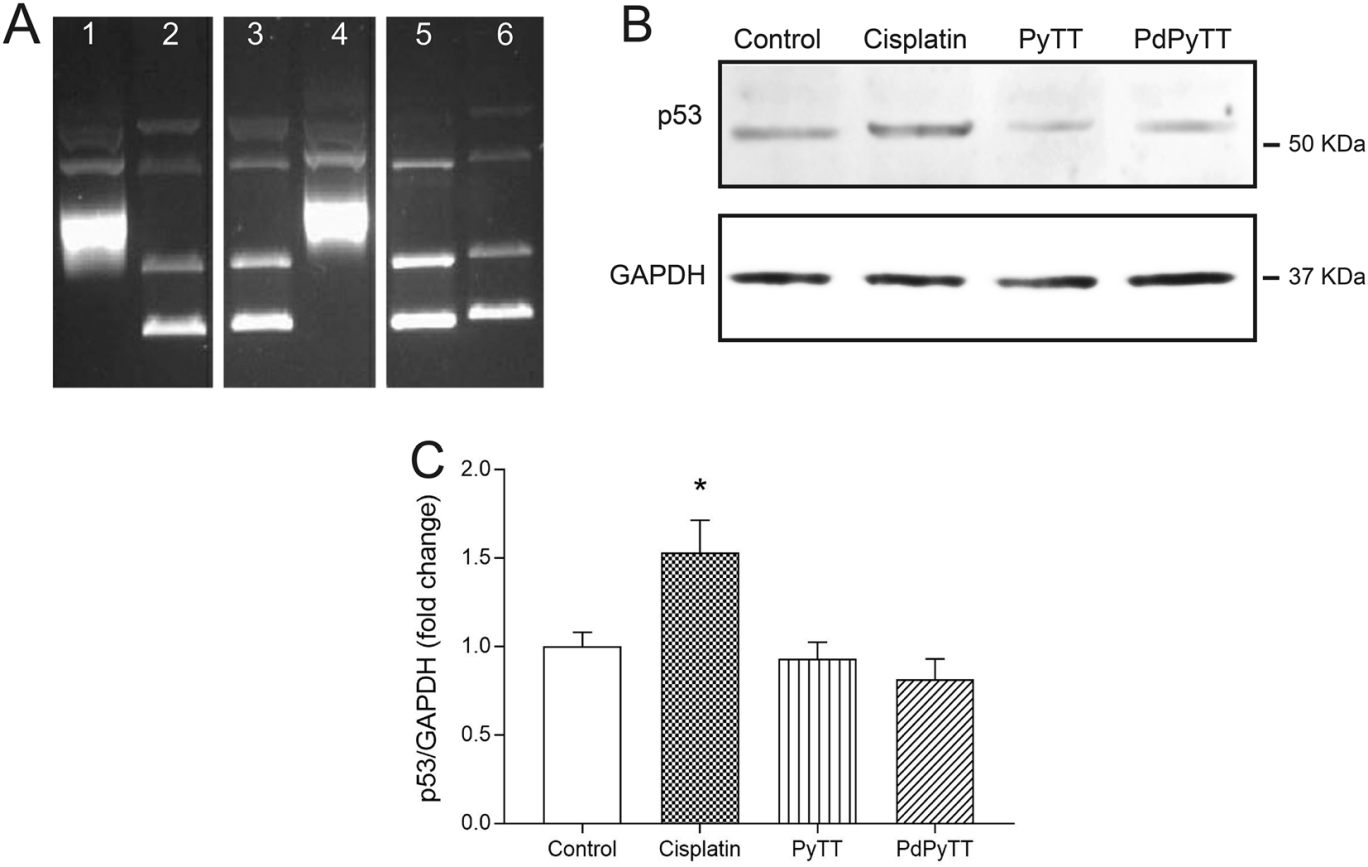
The complex PdPyTT lacks DNA intercalating capacity. (**A**) DNA intercalation was assessed by means of a DNA unwinding assay kit, as explained in Materials and methods. Lane 1: supercoiled pBR322; lane 2: supercoiled pBR322 + wheat germ Topoisomerase I; lane 3: supercoiled pBR322 + wheat germ Topoisomerase I + 20.7 µM PdPyTT; lane 4: supercoiled pBR322 + 20.7 µM PdPyTT; lane 5: relaxed pBR322; lane 6: relaxed pBR322 + wheat germ Topoisomerase I. If a given compound were an intercalating agent, it would unwind the supercoiled DNA or displace topoisomerase I from the groove, thus producing DNA mobility shifts which is not the case for the complex PdPyTT. (**B**) Western blot analysis of whole-cell lysates to detect p53 expression in HL-60 cell line 24 h after treatment with 11.3 µM cisplatin, 20.7 µM PyTT, 20.7 µM PdPyTT, or the vehicle (DMSO, control). GAPDH levels were used as loading controls. For the shake of clarity, gels/blots were cropped and merged in a single image as they were obtained from different parts of the same gel/membrane. Full-length gels and blots are presented in Supplementary Figures S10 and S11, respectively. (**C**) Histograms represent p53 protein levels normalized to GAPDH content and are presented as fold change. Data are presented as mean ± S.D. of 3 independent experiments. *P < 0.05 compared to control values.

**Figure 9:**
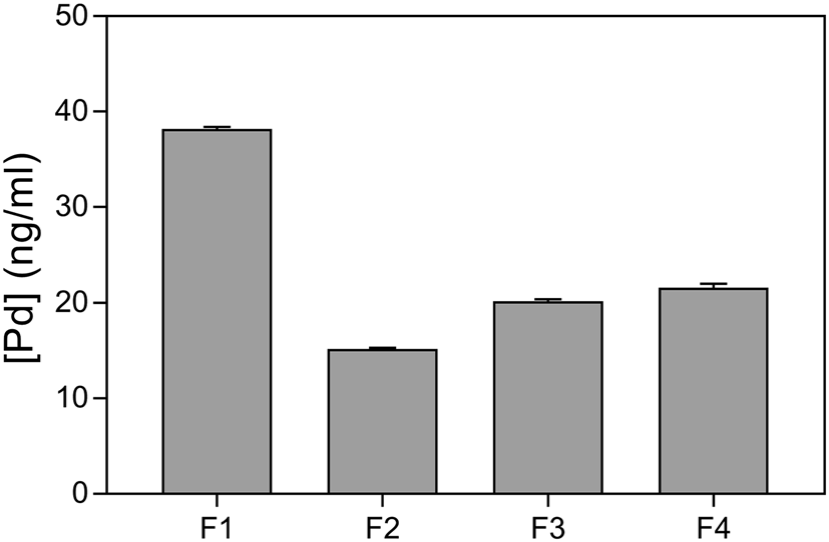
Palladium accumulation in different fractions of HL-60 cells. Cells were treated with 20.7 μM PdPyTT during 5 h, fractionated by means of a ProteoExtract subcellular proteome extraction kit, and then samples were digested with 65% HNO_3_ and subsequently analysed by ICP-MS to quantify metal accumulation. Pd concentrations were given in ng/ml. Values represent means ± S.D. of 3 independent experiments. F1: cytosolic fraction; F2: membrane/organelle fraction; F3: nuclear fraction; F4: cytoskeletal fraction. Pd, hence the complex PdPyTT, was shown to preferentially accumulate in cytosolic fraction.

Finally, we analysed DNA oxidative damage to study its potential contribution to apoptosis of HL-60 treated with the chemotherapeutic agents. Thus, treatment of HL-60 cells with 20.7 µM PdPyTT or 11.3 µM cisplatin for 24 h produced DNA oxidative damage as revealed by the increase in the proportion of cells positively labelled with 8-OHdG, a marker of DNA oxidative damage (Fig. 7). In this case, the effect of both cisplatin and PdPyTT was comparable since there was no significant statistical difference (45.3 ± 18.1 and 34.5 ± 11.9% of positive cells, respectively; Fig. 7A,B), while the free ligand (PyTT) again produced no effect on DNA oxidative damage. Such induction of DNA oxidative damage by both compounds (20–30% increase vs. control) agrees with the induction of mitochondrial ROS production (20% increase vs. control), which may suggest that mitochondrial oxidative stress is specially affecting the mitochondrial pool of DNA as it is extremely susceptible to oxidative damage^44^. Nonetheless, the mechanism of action to trigger the apoptotic process differs amongst both compounds (cisplatin and PdPyTT). In fact, cisplatin is a well-established DNA crosslinking agent that raised p53 (a tumor suppressor that can initiate apoptosis if DNA damage proves to be irreparable) protein expression levels (Fig. 8B,C), while the complex PdPyTT was shown to lack DNA intercalating capacity (Fig. 8A) and did not affect p53 expression (Fig. 8B,C). These findings were also in line with data on cellular accumulation (Fig. 9) as, once internalized, the palladium(II) complex was preferentially found into the cytosol, wherein it triggered oxidative stress (Fig. 5B,C) that subsequently spread to mitochondria (Fig. 5A,C).

Unlike cisplatin and various previously reported palladium(II)-based complexes^45–48^, PdPyTT did not interact with DNA as demonstrated by the inability of the complex to displace DNA-bound ethidium bromide (Fig. 8A), suggesting that cisplatin and PdPyTT-induced apoptosis in HL-60 cells were driven by different mechanisms. In fact, cisplatin-evoked apoptosis was dependent on genotoxic stress and subsequent p53 activation (the observed ROS overproduction was secondary to apoptosis), while PdPyTT caused p53-independent apoptosis through ROS-mediated mitochondrial dysfunction, ultimately leading to DNA oxidative damage. In this sense, previously reported palladium(II)-based complexes were shown to affect the expression of several key proteins involved in both the extrinsic pathway, such as caspase 8 and Fas death receptor^18,42,49^, and the intrinsic pathway, such as Bax and cytochrome *c*^50^. Likewise, some palladium(II)-based complexes have exhibited cytotoxicity against malignant cells that was associated to their prooxidative actions that led to increases in levels of either cytosolic ROS or mitochondrial superoxide anion^18,51,52^.

## Conclusion

Apoptosis induction via the intrinsic pathway is a chief mechanism triggered by different chemotherapeutic agents, including cisplatin^53^. Nevertheless, main limitations of canonical chemotherapy treatment are derived from undesirable side effects and development of resistance during treatment. In this context, new organometallic drugs acting through nonconventional mechanisms and with lower off target toxicity are necessary to improve therapeutic efficiency.

Palladium(II)-based complexes represent an appealing alternative to classical antitumor drugs as they possess promising chemotherapeutic properties including covalent interaction with nitrogen bases of DNA, activation of either the extrinsic or the intrinsic apoptosis pathway, blockade of cell cycle progression, and induction of ROS overproduction that ultimately leads to oxidation of biomolecules^54^. Herein, we have synthesized a thiazoline-based palladium(II) complex that exhibited cytotoxicity towards human promyelocytic leukemia HL-60 cells, while it proved to be innocuous in normal non-cancer cells, such as freshly isolated human leukocytes. Additionally, the palladium(II) complex described in the present study showed pro-apoptotic properties dependent on mitochondrial pathway and intracellular ROS production. Therefore, the complex PdPyTT may be considered as promising candidate for developing new palladium-based antitumor/antileukemic agents, although its safety profile should be further tested in animal models to identify potential side effects.

## Experimental methods

### General procedures

The experimental protocols were approved by the ethics committee of the University of Extremadura. Human promyelocytic leukemia HL-60 15-12 cell line (ECACC No 88120805) and human histiocytic lymphoma U-937 cell line (ECACC No. 85011440) were purchased from The European Collection of Cell Cultures (ECACC) (Dorset, U.K.). RPMI 1640 and penicillin/streptomycin were acquired from HyClone (Aalst, Belgium). CaspGLOW fluorescein active caspase-3 staining kit, foetal bovine serum (FBS), Hoechst 33342, Hoechst 33258, F(ab')2-goat anti-mouse IgG (H + L) secondary antibody Alexa Fluor 633, MitoSox, propidium iodide (PI) and 2′,7′-dichlorodihydrofluorescein diacetate (DCFH-DA) were purchased from ThermoFisher Scientific (Barcelona, Spain). L-Glutamine was obtained from Lonza (Basel, Switzerland). *Cis*-diammineplatinum(II) dichloride (cisplatin), agarose, ethidium bromide, saponin, paraformaldehyde (PFA) and anti-glyceraldhehyde-3-phospate dehydrogenase (GAPDH) mouse monoclonal antibody (clone 6C5) were bought from Sigma Aldrich (Madrid, Spain). Caspase-9 staining kit (Red) was purchased from Abnova (Taipei, Taiwan). CellTiter 96 AQueous One Solution Cell Proliferation Assay was acquired from Promega (Madrid, Spain). anti-8-hydroxy-2′-deoxyguanosine (8-OHdG) mouse monoclonal antibody (clone N45.1) was obtained from JaICa (Fukuroi, Japan). Anti- p53 rabbit polyclonal antibody (clone FL-393) was bought from Santa Cruz Biotechnology (Heidelberg, Germany). Leuko Spin medium was obtained from pluriSelect (Leipzig, Germany). All other reagents were of analytical/commercial grade and were used without further purification.

Elemental analyses (C, H, N and S) were carried out on a Leco CHNS-932 microanalyzer. Infrared spectra were recorded on a Perkin-Elmer FT-IR 1720 spectrophotometer, from KBr pellets (4000–370 cm^-1^) and on a Perkin-Elmer FT-IR 1700X spectrophotometer, from Nujol mulls (500–150 cm^-1^). ^1^H NMR spectra were obtained with a Bruker Avance 500 instrument at 500 MHz in DMSO-*d*_*6*_. ESI–MS was carried out with an Agilent Q-TOF 6520 instrument with the sample solved in DMSO and using water (0.1% formic acid) as mobile phase.

### Synthesis of 2-(2-pyridil)imino-N-(2-thiazolin-2-yl)thiazolidine (PyTT)

Ligand was synthesized following a procedure previously published^55^, with minor modifications as described in literature^56^. ^1^H NMR (500 MHz, DMSO-*d*_6_) δ 8.36 (dd, *J* = 5.0, 1.9 Hz, 1H), 7.75 (td, *J* = 7.7, 2.0 Hz, 1H), 7.09–7.04 (m, 2H), 4.15 (t, *J* = 7.3 Hz, 2H), 3.90 (t, *J* = 8.1 Hz, 2H), 3.21 (t, *J* = 7.3 Hz, 2H), 3.15 (t, *J* = 8.1 Hz, 2H).

### *Synthesis of [PdCl*_*2*_*(µ-PyTT)*_*2*_*]Cl*_*2*_*·4H*_*2*_*O (PdPyTT)*

A solution of Na_2_[PdCl_4_]·H_2_O (157 mg, 0.5 mmol) in ethanol (5 ml) was mixed with an ethanol solution (30 ml) of PyTT (166 mg, 0.5 mmol), refluxed for 24 h and then cooled at room temperature. The resulting orange solid was filtered off, resulting in a mixture of substance where we did not purify any compound. The filtered solution was allowed to evaporate at room temperature. After 24 h, orange crystals suitable for X-ray diffraction analysis were isolated from the solution by filtration (230 mg, 45%). Orange solid turned out to be a mixture of compounds that could not be separated by recrystallization or chromatography. Anal. Calc. (%) for C_22_H_24_Cl_4_N_8_Pd_2_S_4_·4H_2_O: C, 27.65; H, 3.37; N, 11.37; S, 13.42%. Found: C, 27.62; H, 3.22; N, 11.73; S, 13.09%. IR (KBr): 3084, 2933, 2858 ν(C-H), 1601 ν(C=N), 1530, 1470, 1429, 1384, 1014, 631, (pyridine ring vibrations), 1601, 808, 631, 529, 430 (thiazoline ring vibrations), 1032, 886, 700, (thiazolidine ring vibrations), 325, 285, 254, 214 cm^-1^ (metal–ligand vibrations). ^1^H NMR (500 MHz, DMSO-*d*_6_) δ 8.38 (dd, *J* = 5.2, 1.8 Hz, 1H), 7.77 (td, *J* = 7.7, 2.0 Hz, 1H), 7.11–7.06 (m, 2H), 4.17 (t, *J* = 7.3 Hz, 2H), 3.92 (t, *J* = 8.2 Hz, 2H), 3.23 (t, *J* = 7.3 Hz, 2H), 3.18 (t, *J* = 8.2 Hz, 2H). ESI–MS 406.925 [M-H]^2+^.

### Crystal structure determination

X-ray diffraction data were collected on a Bruker Kappa APEXII CCD diffractometer with Mo Kα radiation (λ = 0.71073 Å). Absorption corrections were applied using the program SADABS^57^. Using WINGX^58^, the structures were solved by direct methods (SHELXS-14^59^) and refined by full-matrix least-squares on F^2^ (SHELXL-18^60^). All non-hydrogen atoms were refined using anisotropic displacement parameters. Hydrogen atoms attached to carbon atoms were located in calculated positions and were refined using a riding model. However, hydrogen atoms of the water molecules were detected by Fourier differences and were refined with fixed O–H and H–H distances (0.957(3) and 1.513(3) Å, respectively). Graphical representations of the molecular structures were generated using ORTEP3^58^. CCDC 1988539 contains the supplementary crystallographic data for PdPyTT (Cambridge Crystallographic Data Center; copies of the data can be obtained, e-mail: deposit@ccdc.cam.ac.uk or www: https://www.ccdc.cam.ac.uk). Experimental details of the crystal structure determination are listed in Table 2.

**Table 2:** Crystal data, data collection and refinement details for PdPyTT.

	PdPyTT
Crystal shape	Prism
Color	Orange
Size (mm)	0.15 × 0.15 × 0.13
Chemical formula	C_22_H_32_Cl_4_N_8_O_4_Pd_2_S_4_
Formula weight	955.39
Crystal system	Monoclinic
Space group	P 21/c
**Unit cell dimensions**	
*a* (Å)	14.184(5)
*b* (Å)	21.669(8)
*c* (Å)	21.746(8)
*β* (º)	90.63(2)
Cell volume (Å^3^)	6683(4)
Z	8
D_calc_ (g cm^−3^)	1.899
*μ* (mm^−1^)	1.69
F(000)	3808
θ range	1.327–33.142
Index ranges	− 21 ≤ h ≤ 21, 0 ≤ k ≤ 33, 0 ≤ l ≤ 33
Temperature (K)	298
Independent reflections	25,449
Observed reflections	21564[F > 4.0 σ(F)]
Max/min transmission	0.8376/0.7716
No. of refined parameters	857
*R* [F > 4.0 σ(F)]	0.0333
*wR* [F > 4.0 σ(F)]	0.0644
GOF	1.176
*ρ*_*max*_*, ρ*_*min*_ (e Å^−3^)	0.698, − 1.145

### Cell culture and treatments

HL-60 and U-937 cells were grown in RPMI 1640 medium supplemented with 2 mM L-glutamine, 10% heat-inactivated foetal bovine serum, 100 U/ml penicillin and 100 µg/ml streptomycin at 37ºC under a humidified condition of 95% air and 5% CO_2_. Cells were routinely plated at a density of 3 × 10^5^ cells/ml into fresh flasks and the viability was routinely kept at > 95% as assayed by the Trypan-blue exclusion method. Cells were treated with the free ligand PyTT, the thiazoline-containing palladium(II) complex PdPyTT, cisplatin (positive control) or vehicle for 24 h at the indicated concentrations. Dimethyl sulfoxide (DMSO) was used as vehicle and its final concentration did not exceed 0.1% (v/v).

### Blood collection and isolation of human leukocytes

Venous blood was drawn from healthy volunteers aged 20–45 years. All volunteers gave informed consent. The procedure was carried out according to local ethical committee guidelines and the Declaration of Helsinki. In brief, leucocytes were separated from whole blood by using Leuko Spin medium. Samples were centrifuged at 1000 × g for 30 min at room temperature and then leukocyte cell fraction was isolated from the medium/plasma interface. Leukocytes were washed twice in phosphate-buffered saline (PBS) at 300 × g for 10 min at 4 °C, the supernatant was removed, and the cell pellet was finally re-suspended in RPMI-1640 medium.

### In vitro cytotoxicity assay

The cytotoxic effects of the different compounds were assayed on human promyelocytic leukemia HL-60 cell line and human leukocytes by means of the CellTiter 96 AQueous One Solution Cell Proliferation Assay, which is based on the reduction of an MTS tetrazolium compound. Cells were seeded in 96-well plates at a density of 2 × 10^4^ cells/well. After treating cultures for 24 h, assays were performed by adding 10 µl of the CellTiter 96 AQueous One Solution Reagent directly to culture wells, incubating for 2 h (for HL-60 cells) or 4 h (for leukocytes) at 37 °C, and then recording absorbance on a microplate reader (Infinite M200; Tecan) at both a test wavelength of 490 nm and a reference wavelength of 650 nm to subtract background. All analyses were run in triplicate. The cell viability was calculated as percentage of control values (untreated samples).

### Determination of apoptosis

To evaluate apoptosis, the fraction of apoptotic nuclei was quantified by fluorescence microscopy after DNA staining with Hoechst 33342. The dye was used at a final concentration of 1 µg/ml. Then, at least 100 cells per field were counted at the epifluorescence microscope (Nikon Eclipse TS100 equipped with a LED illumination system CoolLED pE-300 white and a digital camera DS-Qi1Mc) in at least three randomly selected microscopic fields. Hoechst 33342 was excited using a single-band filter set, optimized for DAPI and other like fluorophore (Semrock). The images were analysed with the macro “Apoptosis and Cell Density Macro” available in the open source image processing package Fiji/ImageJ (https://imagejdocu.tudor.lu/plugin/morphology/apoptosis_and_cell_count_macro/start) and percentage of apoptotic cells was computed.

Alternatively, induction of apoptosis was determined by analysing phosphatidylserine externalization via annexin V-FITC/PI assay (ThermoFisher Scientific). Briefly, stimulated cells were harvested (4 × 10^5^ cells/ml), washed once with PBS, and centrifuged at 300 × g for 5 min; then, the supernatant was discarded, and the pellet was resuspended in 200 µl binding buffer containing 5 µl of annexin V-FITC. Cells were incubated for 10 min at room temperature, washed once with binding buffer, and finally resuspended in 200 µl binding buffer containing 10 µL of PI. Stained cells were immediately analysed by a MACSQuant X flow cytometer (Miltenyi Biotech). Annexin V-FITC was excited with 488 nm laser and its fluorescence was acquired through 525/50 nm BP filter, while PI was excited with 488 nm laser and its fluorescence was acquired through 585/40 nm BP filter. Ten thousand events/cells were analysed per condition. Each sample was tested six times in independent experiments. The sum of early apoptosis and late apoptosis was calculated to obtain the total percentage of apoptotic cells^61,62^.

### Caspase-3 and -9 analysis

Cells were seeded in 6-well plates at a density of 2 × 10^6^ cells/well. After treating cultures for 24 h, caspase-3 and -9 activation was measured with CaspGLOW fluorescein active caspase-3 staining kit and Caspase-9 staining kit (Red), respectively. Flow cytometry analysis was performed using a MACSQuant Analyzer 10 flow cytometer (Miltenyi Biotech) equipped with 3 lasers (405, 488 and 561 nm). CaspGLOW fluorescein was excited with 488 nm laser and its fluorescence was acquired through 525/50 nm BP filter, while Red-LEHD-FMK (fluorescent marker for caspase-9) was excited with 561 nm laser and their fluorescence was acquired through 586/15 nm BP filter. Analysis was restricted to live cells that were detected by using the vital dye Hoechst 33,258 (10 µg/ml), which was excited with 405 nm laser and its fluorescence acquired through 450/50 nm BP filter. Ten thousand events/cells were analysed per condition. Each sample was tested six times in independent experiments. Data were presented as percentage of stained cells.

### Measurement of reactive oxygen species (ROS)

Cells were seeded in 6-well plates at a density of 2 × 10^6^ cells/well. After treating cultures for 24 h, assays were performed by incubating cells (30 min, 37 °C) with 0.4 µM 2′,7′-dichlorodihydrofluorescein diacetate (DCFH-DA) or 500 nM MitoSox, which allow to determine intracellular reactive oxygen species (ROS) overproduction and mitochondrial superoxide anion generation, respectively. Flow cytometry analysis was performed using a MACSQuant Analyzer 10 flow cytometer (Miltenyi Biotech). DCF was excited with 488 nm laser and its fluorescence was acquired through 525/50 nm BP filter, while MitoSox was excited with 561 nm laser and its fluorescence was acquired through 586/15 nm BP filter. Again, analysis was restricted to live cells that were detected by using the vital dye Hoechst 33258 (10 µg/ml), which was excited with 405 nm laser and its fluorescence acquired through 450/50 nm BP filter. Ten thousand events/cells were analysed per condition. Each sample was tested six times in independent experiments. Data were presented as percentage of stained cells.

### Cell cycle analysis

Cells were seeded in 6-well plates at a density of 2 × 10^6^ cells/well. After treatment, cells were washed with PBS and fixed in 70% ethanol for 30 min at 4 °C. The cells were again rinsed with PBS and resuspended in 500 ml of PBS containing PI (20 µg/ml) and RNase A (50 µg/mL). The samples were kept in the dark at 4 °C for 30 min and then stained cells were immediately analysed by a MACSQuant X flow cytometer (Miltenyi Biotech)^63^. PI was excited with 488 nm laser and its fluorescence was acquired through 585/40 nm BP filter. Ten thousand events/cells were analysed per condition. Each sample was tested six times in independent experiments. Doublets were removed from the analysis by including a single cells gate in the acquisition protocol^64^.

### Detection of DNA oxidative damage

Cells were seeded in 6-well plates at a density of 2 × 10^6^ cells/well. After treating cultures for 24 h, cells were washed twice with PBS and then fixed with PFA (4% in PBS, pH 7.4) for 30 min at room temperature (RT). Fixed cells were washed once with PBS, permeabilized with 0.1% saponin (RT, 15 min) and incubated in darkness (1 h at RT) with 1 µg/ml of anti-8-OHdG antibody, which is a marker of DNA oxidative damage. At the end of incubation, cells were washed once with PBS and then incubated in darkness (1 h at RT) with 8 µg/ml of F(ab')2-goat anti-mouse IgG (H + L) secondary antibody, Alexa Fluor 633. Afterwards, cells were washed once, re-suspended with PBS and stained with PI in order to distinguish nucleated cells. Flow cytometry analysis was performed using a MACSQuant X flow cytometer (Miltenyi Biotech). PI was excited with 488 nm laser and its fluorescence was acquired through 585/40 nm BP filter, while Alexa Fluor 633 was excited with 640 nm laser and its fluorescence was acquired through 660/20 nm BP filter. Ten thousand events/cells were analysed per condition. Each sample was tested six times in independent experiments. Data were presented as percentage of stained cells.

### Western blot

HL-60 cells were grown on 10 cm Petri dishes and challenged with the indicated compounds. After treatments, cells were washed twice with PBS, re-suspended in RIPA buffer (150 mM NaCl, 50 mM Tris-Cl pH 7.4, 1% Triton-X100, 0.5% sodium deoxycholate, 0.1% SDS and Complete EDTA-free protease inhibitor mixture) and briefly sonicated. Crude extracts were centrifuged at 15,000 × g for 10 min to remove debris, and proteins in the supernatant were then quantified using the BCA Protein Assay Kit (ThermoFisher Scientific). Forty μg of proteins were dissolved in LDS sample buffer, heated for 10 min at 70 °C and loaded on 4–12% Bis-Tris NuPage gels. After electrophoretic separation, proteins were transferred onto nitrocellulose membranes and probed with the indicated primary antibodies. Isotype matched horseradish peroxidase-conjugated secondary antibodies were used following by detection by chemiluminescence (Super-Signal West Dura). Densitometry was performed using the built-in function present in the image processing package Fiji/ImageJ^65^. For the shake of clarity, full-length blots (Supplementary Figure S11) were cropped and merged in a single image (Fig. 8) with the software package Adobe Illustrator CS6 as they were obtained from different parts of the same membrane.

### DNA intercalation assay

DNA intercalation was assessed by examining the ability of the complex PdPyTT to displace ethidium bromide from supercoiled and/or relaxed pBR322 plasmid. To do so, DNA unwinding assay kit (Inspiralis) was used following manufacturer’s instructions. Briefly, 0.5 µg of DNA (supercoiled or relaxed pBR322) were added to 15 µl of 2 × assay buffer (50 mM Tris-HCl (pH 7.9), 1 mM EDTA, 1 mM DTT, 20% (v/v) glycerol, 50 mM NaCl) and then incubated with 20.7 µM PdPyTT or the vehicle (negative control) for 5 min at RT. Afterwards, 2 µl of 1 × dilution buffer (50 mM Tris-HCl (pH 7.9), 1 mM EDTA, 1 mM DTT, 50% (v/v) glycerol, 500 mM NaCl) and 2 µl of wheat germ Topoisomerase I were added to initiate unwinding and samples were incubated at 37 °C for further 30 min. Reactions were terminated by addition of 20 µl of water and 50 µl of butanol. Reactions were separated on 1% (w/v) agarose gel in 1 × TBE (Tris–borate-EDTA) buffer and gel was run at 80 V for approximately 4 h, stained with ethidium bromide, and visualised with a gel documentation system (ChemiDoc XRS + System, Bio-Rad). For the shake of clarity, full-length gels (Supplementary Figure S10) were cropped and merged in a single image (Fig. 8) with the software package Adobe Illustrator CS6 as they were obtained from different parts of the same gel.

### Cellular uptake by ICP-MS

To quantify the metal accumulation in different fractions of the cells, HL-60 cells were seeded in culture flasks (2 × 10^6^ cells in 5 ml RPMI-1640 culture medium). After 24 h in culture, cells were treated for 5 h with 20.7 µM of PdPyTT, were then harvested and subcellular fractionation was performed to obtain different cell fractions: F1: cytosolic fraction; F2: membrane/organelle fraction; F3: nuclear fraction; F4: cytoskeletal fraction. In this sense, cells were fractionated by means of a ProteoExtract subcellular proteome extraction kit. Afterwards, the different samples were digested with 65% HNO_3_ at room temperature and analysed by inductively coupled plasma mass spectrometry (ICP-MS 7900, Agilent Technologies).

### Statistical analysis

Data are presented as mean ± standard deviation (S.D.). To compare the different treatments, statistical significance was calculated by one-way analysis of variance (ANOVA) followed by Dunnett’s test. The IC_50_ of the dose–response curve of each compound was calculated by fitting the curve to the data using nonlinear regression to generate a four-parameter sigmoid dose–response equation. *P* < 0.05 was considered to indicate a statistically significant difference. The statistics software used was GraphPad Prism 7.04 for Windows.

### Data availability

The datasets generated during the current study are available from the corresponding authors on request.

## Supplementary information

Supplementary Information 1.
