# Supplementary material for: Synthesis and structure of a new thiazoline-based palladium(II) complex that promotes cytotoxicity and apoptosis of human promyelocytic leukemia HL-60 cells
Source: Sci Rep. 2020 Oct 7;10:16745. doi: 10.1038/s41598-020-73488-0 (PMC7542172; doi:10.1038/s41598-020-73488-0)
Supplement: Supplementary file 1 — Supplementary Information 1. [file 41598_2020_73488_MOESM1_ESM.docx]

Supplementary information for:

**Synthesis and structure of a new thiazoline-based palladium(II) complex that promotes cytotoxicity and apoptosis of human promyelocytic leukemia HL-60 cells.**

***Javier Espino^1^, Elena Fernández-Delgado^1^, Samuel Estirado^1^, Felipe de la Cruz-Martinez^2^, Sergio Villa-Carballar^1^, Emilio Viñuelas-Zahínos^2^, Francisco Luna-Giles^2^, José A. Pariente^1^***

*^1^* *Department of Physiology (Neuroimmunophysiology and Chrononutrition Research Group), University of Extremadura, 06006, Badajoz, Spain;*

*^2^* *Department of Organic and Inorganic Chemistry (Coordination Chemistry Group), University of Extremadura, 06006, Badajoz, Spain*

* Corresponding author: José A. Pariente, [pariente@unex.es](mailto:pariente@unex.es)


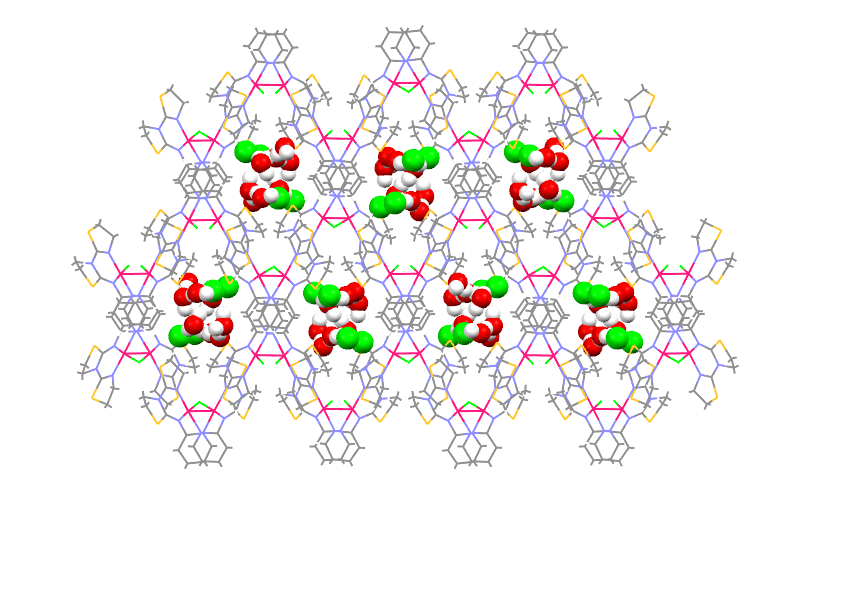


**Figure S1.** *3D supramolecular metal organic matrix along a-axis in PdPyTT.*


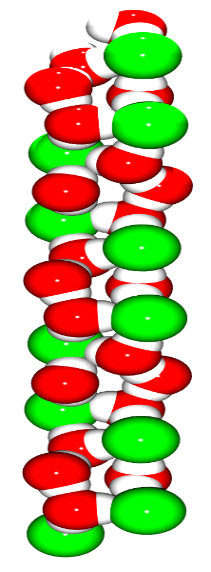

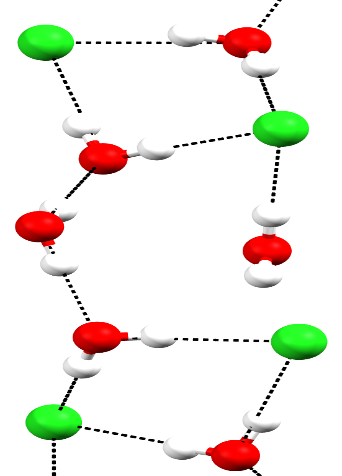


**Figure S2.** *Hydrogen bonds inside water-chloride chains in PdPyTT.*


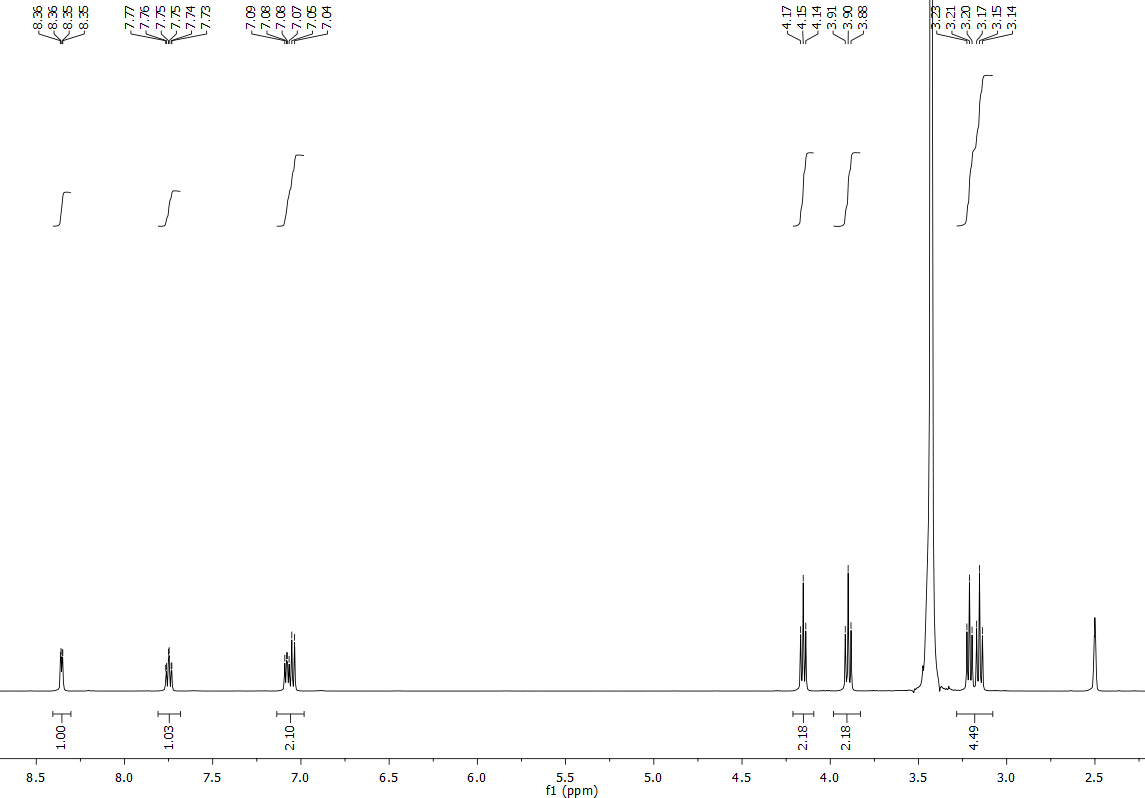


**Figure S3.** *^1^H NMR spectrum of PyTT in DMSO-d_6_.*


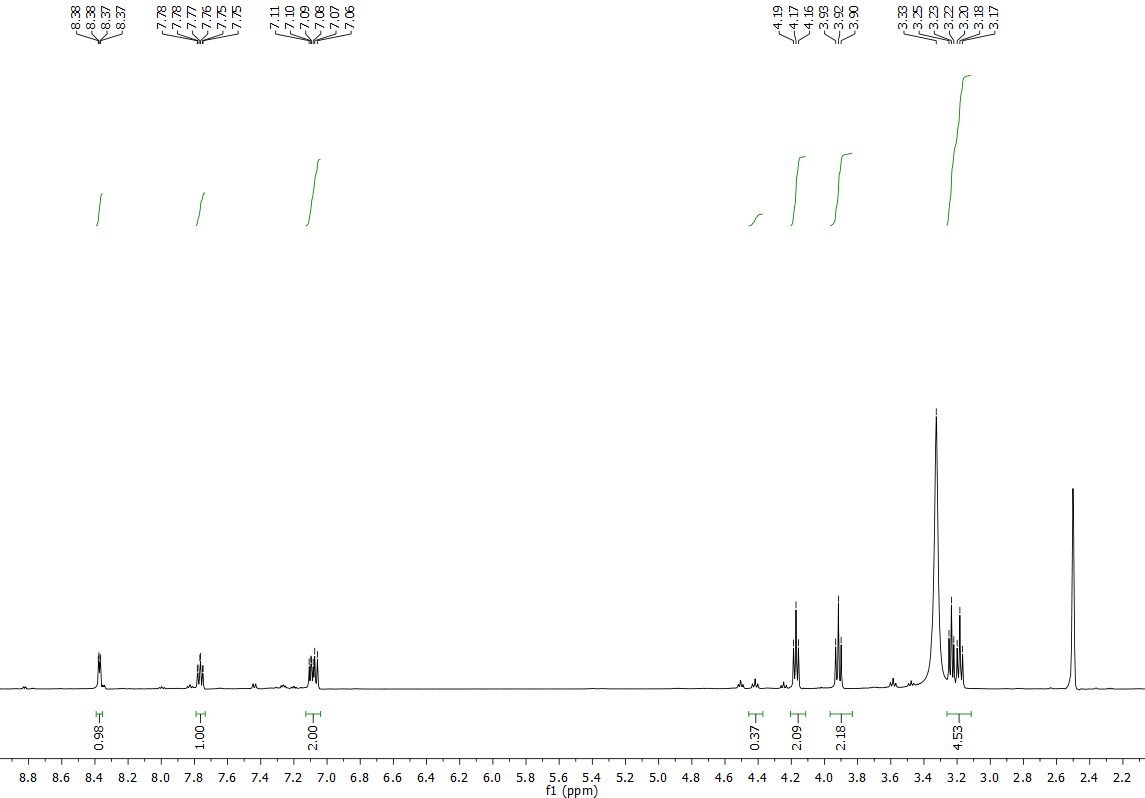


**Figure S4.** *^1^H NMR spectrum of [Pd_2_Cl_2_(PyTT)_2_]Cl_2_·4H_2_O in DMSO-d_6_.*

**Figure S5.** *IR spectrum of [Pd_2_Cl_2_(PyTT)_2_]Cl_2_·4H_2_O in 4000-400 cm^-1^ region.*

**Figure S6.** *IR spectrum of [Pd_2_Cl_2_(PyTT)_2_]Cl_2_·4H_2_O in 500-150 cm^-1^ region.*

**
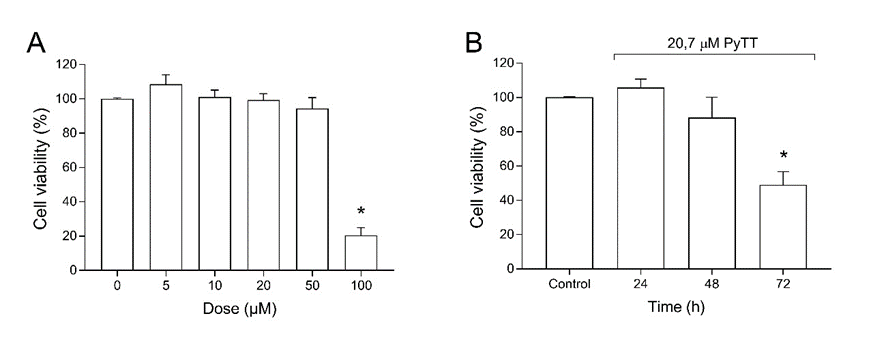
**

**Figure S7**: *Dose- and time-response curves of the palladium complex ligand on cell viability in HL-60 cells. (A) Cells were treated for 24 hours with increasing concentrations (5, 10, 20, 50 and 100 µM) of free ligand (PyTT), or the vehicle (DMSO, control). (B) Cells were treated with 20.7 µM PyTT, or the vehicle (DMSO, control) for 24, 48 and 72 hours. Cell viability was evaluated by means of the MTS assay. Values are presented as means ± S.D. of 6 independent experiments and expressed as percentage of control values (untreated cells). *P<0.05 compared to control values.*


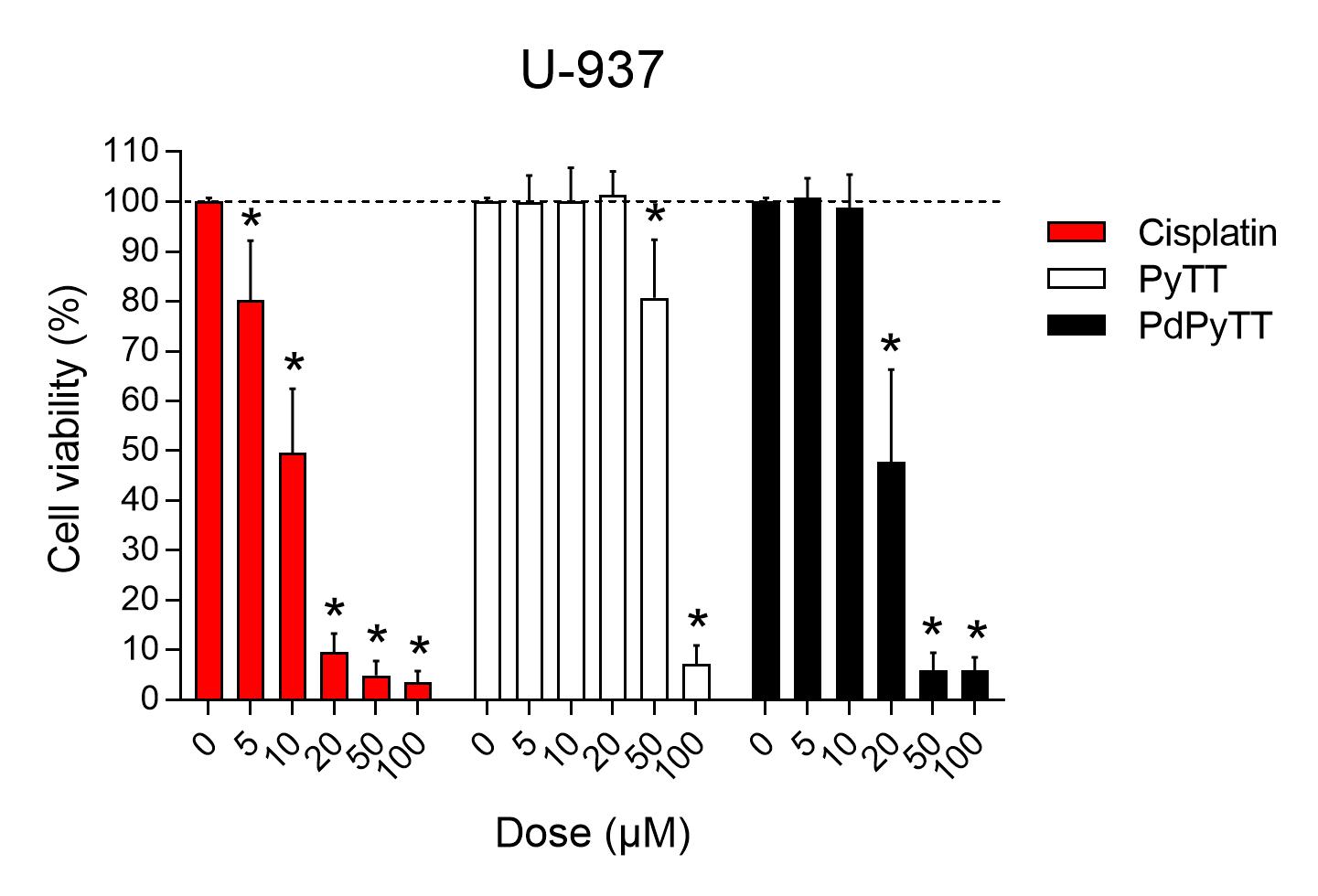


**Figure S8:** *Dose-response curve of chemotherapeutics on cell viability of U-937 cells. (A) U-937 cells were treated for 24 hours with increasing concentrations (5, 10, 20, 50 and 100 µM) of cisplatin, the free ligand PyTT, the Pd(II) complex (PdPyTT), or the vehicle (DMSO, control). Values represent means ± S.D. of 6 independent experiments and are expressed as percentage of control values. *P<0.05 compared to their corresponding control values.*


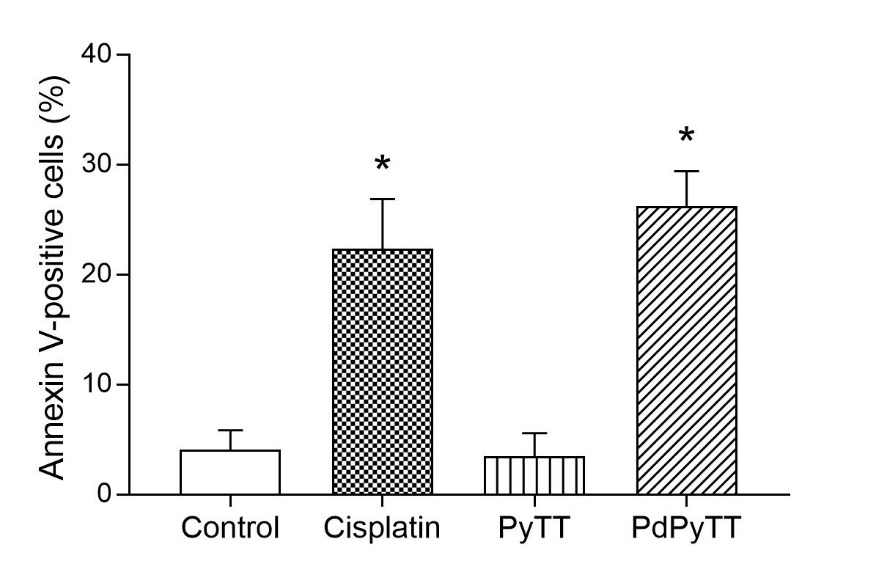


**Figure S9:** *Effects of chemotherapeutics on phosphatidylserine externalization in HL-60 cells. Cells were treated with 11.3 µM cisplatin, 20.7 µM PyTT, 20.7 µM PdPyTT, or the vehicle (DMSO, control) for 24 hours. The phosphatidylserine externalization was determined by double-staining cells with annexin V-FITC/PI and analysed by flow cytometry. As indicated in material and methods, annexin V-positive cells were considered apoptotic cells.* *Histograms show percentages of apoptotic cells. Values represent means ± S.D. of 6 independent experiments. *P<0.05 compared to control values.*


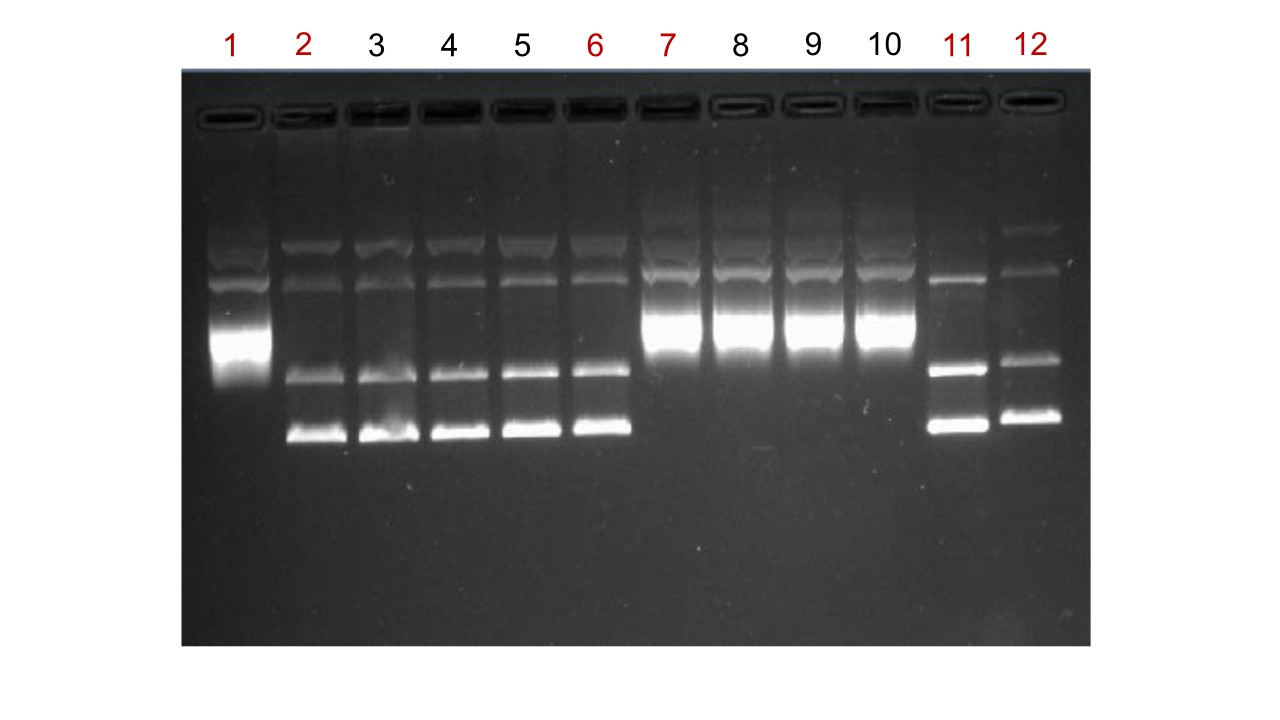


**Figure S10:** *Full-length agarose gel* *corresponding to Figure 8A. Lane 1: supercoiled pBR322; lane 2: supercoiled pBR322 + wheat germ Topoisomerase I; lane 6: supercoiled pBR322 + wheat germ Topoisomerase I + 20.7 µM PdPyTT; lane 7: supercoiled pBR322 + 20.7 µM PdPyTT; lane 11: relaxed pBR322; lane 12: relaxed pBR322 + wheat germ Topoisomerase I. All other lanes (3-5, 8-10) correspond to different organometallic complexes that are still under evaluation in our lab for their potential therapeutic effects.*


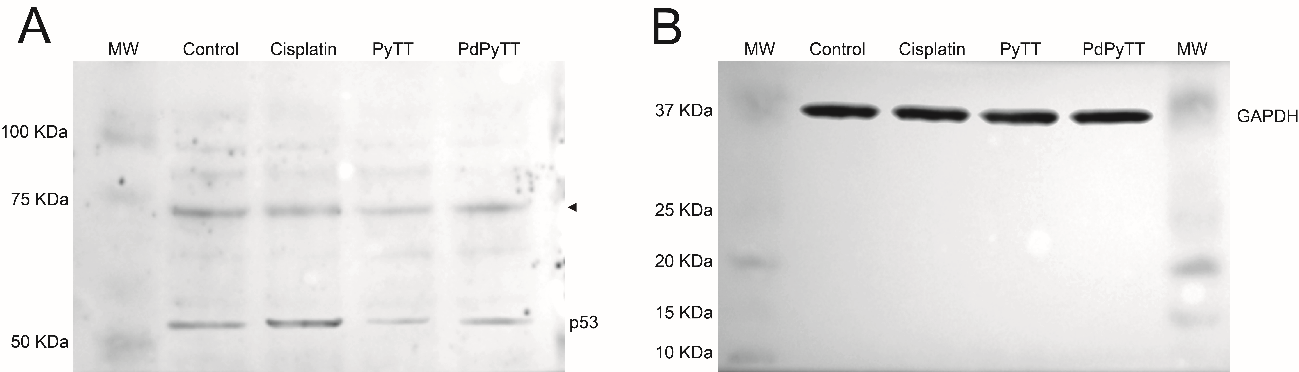


**Figure S11:** *Full-length blots* *corresponding to Figure 8B. p53 (A) and GAPDH (B) protein expression levels. Arrowheads indicate unspecific bands. MW: molecular weight marker.*

**Table S1.** *Hydrogen-bond parameters for PdPyTT*

| D-H ··· A | Position of A | H···A | A-D (Å) | A ··· H-D (º) |
| --- | --- | --- | --- | --- |
| Hydrogen bonds inside water-chloride chain | | | | |
| O(1W)-H(1W)···Cl(5) | -x+2,-y+1,-z+1 | 2.27(2) | 3.226(2) | 178(1) |
| O(1W)-H(2W)···Cl(7) | -x+1,-y+1,-z+1 | 2.39(3) | 3.313(2) | 163(2) |
| O(2W)-H(4W)···Cl(6) | -x,-y,-z+1 | 2.25(2) | 3.165(2) | 161(1) |
| O(2W)-H(3W)···Cl(8) | x-1,y,z | 2.39(3) | 3.293(2) | 158(2) |
| O(3W)-H(5W)···Cl(6) | -x+1,y+1/2,-z+1/2 | 2.28(3) | 3.234(2) | 175(2) |
| O(3W)-H(6W)···Cl(7) | x,y,z | 2.26(3) | 3.172(2) | 158(2) |
| O(4W)-H(8W)···Cl(6) | -x+1,y+1/2,-z+1/2 | 2.23(4) | 3.173(2) | 169(3) |
| O(4W)-H(7W)···Cl(7) | x,y,z | 2.202(7) | 3.144(3) | 168.4(4) |
| O(5W)-H(10W)···Cl(5) | x,-y+1/2,z+1/2 | 2.26(3) | 3.216(3) | 175(2) |
| O(5W)-H(9W)···Cl(8) | x,y,z | 2.21(2) | 3.162(2) | 170(2) |
| O(6W)-H(12W)···Cl(5) | x,-y+1/2,z+1/2 | 2.23(4) | 3.183(3) | 171(3) |
| O(6W)-H(11W)···Cl(8) | x,y,z | 2.23(2) | 3.129(3) | 156(1) |
| O(7W)-H(14W)···O(4W) | x,-y+1/2,z+1/2 | 1.90(2) | 2.838(3) | 167(2) |
| O(7W)-H(13W)···O(5W) | x-1,y,z | 1.92(3) | 2.849(3) | 163(3) |
| O(8W)-H(15W)···O(3W) | x,y,z | 1.90(3) | 2.831(4) | 164(3) |
| O(8W)-H(16W)···O(6W) | x,-y+1/2,z-1/2 | 1.89(2) | 2.844(4) | 177(2) |
| Interactions between water-chloride chain and cationic complexes | | | | |
| C(8)-H(8)···Cl(5) | x-1,-y+1/2,z+1/2 | 2.795(1) | 3.551(3) | 139.2(2) |
| C(8A)-H(8A)···Cl(6) | x,-y+1/2,z+1/2 | 2.693(1) | 3.469(3) | 141.4(2) |
| C(3C)-H(3C1)···Cl(6) | -x+1,-y,-z+1 | 2.777(1) | 3.625(3) | 146.5(2) |
| C(3B)-H(3B1)···Cl(6) | -x+1,-y,-z+1 | 2.924(1) | 3.674(3) | 135.0(2) |
| C(11B)-H(11B)···Cl(7) | x,y,z | 2.843(1) | 3.618(3) | 141.6(2) |
| C(6A)-H(6A1)···Cl(7) | -x+1,-y+1,-z+1 | 2.741(1) | 3.508(3) | 136.4(2) |
| C(6)-H(6A)···Cl(8) | x-1,y,z | 2.659(1) | 3.431(3) | 136.8(2) |
| C(11C)-H(11C)···Cl(8) | -x+2,-y,-z+1 | 2.708(1) | 3.504(3) | 144.1(2) |
| C(3A)-H(3A1)···O(6W) | x-1,-y+1/2,z-1/2 | 2.590(2) | 3.325(4) | 132.7(2) |
| C(11)-H(11)···O(6W) | -x+1,+y+1/2,-z+1/2 | 2.757(2) | 3.553(3) | 144.2(2) |
| C(3A)-H(3A2)···O(1W) | -x+1,-y+1,-z+1 | 2.702(2) | 3.647(4) | 164.7(2) |
| C(11A)-H(11A)···O(7W) | x,y,z | 2.699(2) | 3.493(3) | 143.9(2) |
| C(5B)-H(5B2)···O(7W) | -x+1,-y,-z+1 | 2.459(2) | 3.269(3) | 140.8(2) |
| C(11A)-H(11A)···O(4W) | x,-y+1/2,z+1/2 | 2.686(2) | 3.468(3) | 142.3(2) |
| C(6B)-H(6B2)···O(4W) | -x+1,+y-1/2,-z+1/2 | 2.932(2) | 3.693(4) | 136.1(2) |
| C(11B)-H(11B)···O(4W) | x,y,z | 2.791(2) | 3.570(3) | 142.0(2) |
| C(3C)-H(3C2)···O(4W) | x,-y+1/2,z+1/2 | 2.749(2) | 3.457(4) | 130.3(2) |
| C(5B)-H(5B2)···O(7W) | -x+1,-y,-z+1 | 2.459(2) | 3.269(2) | 140.8(2) |
| C(8B)-H(8B)···O(1W) | x,-y+1/2,z-1/2 | 2.704(2) | 3.462(3) | 139.3(2) |
| C(8B)-H(8B)···O(5W) | -x+2,-y,-z+1 | 2.637(2) | 3.415(3) | 141.5(2) |
| C(5C)-H(5C2)···O(8W) | x,y,z | 2.427(3) | 3.253(4) | 142.8(2) |
| C(8C)-H(8C)···O(2W) | x+1,-y+1/2,z-1/2 | 2.615(2) | 3.308(3) | 131.7(2) |
| C(8C)-H(8C)···O(3W) | x,y,z | 2.626(2) | 3.425(3) | 144.4(2) |
| Interactions between cationic complexes | | | | |
| C(5)-H(5B)···Cl(3) | x-1,y,z | 2.738(1) | 3.518(3) | 138.0(2) |
| C(5A)-H(5A2)···Cl(1) | -x+1,-y+1,-z+1 | 2.779(1) | 3.478(3) | 129.5(2) |
| C(5B)-H(5B1)···Cl(4) | -x+1,-y,-z+1 | 2.789(1) | 3.601(3) | 141.7(2) |
| C(5C)-H(5C1)···Cl(2) | x+1,y,z | 2.735(1) | 3.345(3) | 121.5(2) |
| C(10)-H(10)···Cl(8) | -x+1,y+1/2,-z+1/2 | 2.970(1) | 3.683(3) | 134.6(2) |
| C(10A)-H(10A)···Cl(4) | x,-y+1/2,z+1/2 | 2.955(1) | 3.595(3) | 127.3(2) |
| C(10B)-H(10B)···Cl(1) | x,-y+1/2,z-1/2 | 2.813(1) | 3.604(3) | 143.6(2) |
| C(10C)-H(10C)···Cl(2) | x+1,-y+1/2,z-1/2 | 2.797(1) | 3.621(3) | 148.2(2) |
| C(9)-H(9)···S(1) | x,-y+1/2,z+1/2 | 2.921(1) | 3.627(3) | 133.8(2) |
| C(9B)-H(9B)···S(1A) | -x+1,y-1/2,-z+1/2 | 2.857(1) | 3.491(3) | 126.5(2) |
| C(9B)-H(9B)···S(2A) | x,-y+1/2,z-1/2 | 2.900(1) | 3.480(3) | 121.8(2) |
| C(9C)-H(9C)···S(2) | x+1,-y+1/2,z-1/2 | 2.916(1) | 3.490(3) | 121.3(2) |
